# Resolvin D5 Inhibits CXCL8 Expression in Colonic Epithelial Cells Through Activating GPR101 to Impede Neutrophil Recruitment and Consequently Alleviate Ulcerative Colitis

**DOI:** 10.1002/advs.202515176

**Published:** 2026-02-03

**Authors:** Pengxiang Guo, Yilei Guo, Yanrong Zhu, Ke Yan, Yulai Fang, Jiafeng Zhang, Yue He, Zhifeng Wei, Yufeng Xia, Yue Dai

**Affiliations:** ^1^ Department of Pharmacology of Chinese Materia Medica School of Traditional Chinese Pharmacy China Pharmaceutical University Nanjing China; ^2^ Department of Pharmacognosy School of Traditional Chinese Pharmacy China Pharmaceutical University Nanjing China; ^3^ School of Life Sciences Beijing University of Chinese Medicine Beijing China

**Keywords:** C‐X‐C motif chemokine ligand 8, epimedin A1, G protein‐coupled receptor 101, resolvin D5, ulcerative colitis

## Abstract

In the colonic mucosal tissues of patients with ulcerative colitis (UC), the levels of resolvin D5 (RvD5), a specialized pro‐resolving mediator, are significantly reduced and negatively correlate with disease severity. Whether RvD5 can ameliorate UC remain unclear. Here, we show that exogenous supplement of RvD5 remarkably reduces neutrophil infiltration within the colonic mucosal epithelial layers and alleviates dextran sulfate sodium‐induced colitis in mice. Adoptive transfer of neutrophils significantly attenuates the alleviation of RvD5 against colitis, indicating that inhibition of neutrophil infiltration plays a crucial role in the anti‐colitis effect. Mechanistically, RvD5 activates GPR101 in colonic epithelial cells to selectively downregulate CXCL8 expression through inhibiting pSTAT1 expression and thereby suppressing neutrophil infiltration within colonic mucosal epithelial layer, and GPR101 knockdown attenuates the above‐mentioned effect of RvD5. Based on molecular docking screening from natural product library, epimedin A1 is identified as a novel enhancer of RvD5 biosynthesis in M2 macrophages, which functions through allosteric inhibition of 5‐lipoxygenase (5‐LOX). Epimedin A1 significantly elevates RvD5 levels in colonic tissue, suppresses CXCL8 expression in colonic epithelial cells, and reduces mucosal epithelial neutrophil infiltration in colitis mice. In summary, RvD5 and its biosynthesis promoter epimedin A1 are promising as therapeutic agents of UC.

## Graphical abstract

RvD5 effectively alleviates UC by inhibiting STAT1 signaling to reduce CXCL8 expression in colonic epithelial cells via activation of GPR101, which subsequently decreases the infiltration of neutrophils in the colonic mucosal epithelium. Epimedin A1, a natural allosteric inhibitor of 5‐LOX, demonstrates potential as a therapeutic agent for UC by enhancing the biosynthesis of RvD5 in M2 macrophages.

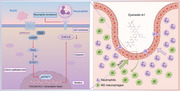

## 1 Introduction

Ulcerative colitis (UC) is a chronic inflammatory disease with complicated etiology, and multiple types of cells are involved in its pathogenesis [1]. Of which, neutrophils significantly contribute to the onset and progression of the disease, especially during the acute phase of pathogen infection and intestinal inflammation [2]. Infiltrated neutrophils in the mucosal epithelium exert potent pro‐inflammatory effects, disrupt epithelial barrier and recruit other immune cells [3, 4, 5]. They are also closely correlated with disease recurrence, treatment resistance, surgical requirements, and colorectal cancer risk [6, 7, 8, 9]. Blockade of neutrophil infiltration drivers, such as CXCR2 and hepatocyte growth factor (HGF), may offer new strategies for UC treatment [10, 11, 12].

Specialized pro‐resolving mediators (SPMs), including resolvins (Rvs), maresins (MaRs), protectins (PDs) and lipoxins (LXs), are biologically active endogenous substances produced by the enzymatic catalysis of ω‐3 and ω‐6 essential fatty acids [13]. Under inflammatory status of UC, the delicate balance between pro‐inflammatory factors, such as cytokines and chemokines, and SPMs is disrupted in mucosal tissues, which affects the homeostasis and repair property of intestinal epithelial cells and finally leads to epithelial injury, ulcers and barrier defects. SPMs synthesized by immune cells and epithelial cells play a crucial role in promoting inflammation resolution and restoring mucosal homeostasis [14]. In experimental colitis induced by dextran sulfate sodium (DSS) and 2,4,6‐trinitrobenzene sulfonic acid (TNBS), several SPMs exhibit clear ameliorative effects [15]. The anti‐inflammatory and immunosuppressive agents commonly used for the treatment of UC often impair the body's response ability to pathogenic microorganisms. As immunomodulators, SPMs do not exhibit such side effect, function through facilitating endogenous processes to aid the body in restoring homeostasis following inflammation, and thereby represent a more meaningful therapeutic approach for UC.

Resolvin D5 (RvD5), derived from docosahexaenoic acid (DHA), is one kind of SPMs with potent pro‐resolving effect [16]. In the colonic mucosal tissues of active UC patients, the levels of RvD5 are significantly reduced. Furthermore, the expression level of RvD5 demonstrates a negative correlation with the severity of UC [17]. Whether and how exogenous supplement of RvD5 ameliorate UC remains to be identified. The present study aims to investigate the beneficial effect of RvD5 on UC in the DSS‐induced murine colitis and elucidate the underlying mechanism by focusing on neutrophil infiltration into the mucosal epithelium. Furthermore, we search for natural products capable of promoting RvD5 biosynthesis as novel candidate agents for UC management.

## 2 Methods

### 2.1 DSS‐Induced Colitis in Mice and Treatments

This study employed female C57BL/6 mice aged 8–10 weeks. All animal experiments were conducted in strict accordance with the protocols approved by the Animal Ethics Committee of China Pharmaceutical University and were granted ethical approval (Approval No.: 2022‐03‐044). To investigate the protective potential of RvD5 on colitis and the influence on neutrophil infiltration into the colonic mucosal epithelium, mice were randomly divided into five groups: normal group, model group, RvD5 (2, 5 µg/kg) group, and 5‐aminosalicylic acid (5‐ASA, 150 mg/kg) group. Apart from the normal group, mice in other groups were allowed free access to a 2.5% DSS solution for seven consecutive days, followed by distilled water for three days. The initiation day of DSS induction was designated as day 1 [18]. The treatment groups received either intraperitoneal injection of RvD5 or oral administration of 5‐ASA for a continuous duration of ten days. At the end of the experiment, the mice were euthanized via cervical dislocation following isoflurane anesthesia, and their colon tissues were harvested.

### 2.2 Adoptive Transfer of Neutrophils

Under sterile conditions, femurs were isolated from littermate mice and placed in sterile cell culture dishes. Using aseptic pipetting, the bone marrow was transferred from the dishes to a cell strainer. Cells were suspended in sorting buffer and incubated on ice for 30 min. Cell counts were performed and adjusted to 1 × 10^8^ cells/mL. 1 mL of cell suspension was taken and 100 µL of Biotin‐Antibody Cocktail was added, followed by incubation on ice for 15 min. Cells were washed with sorting buffer and 100 µL of Streptavidin Nanobeads were added and mixed thoroughly, followed by another 15 min incubation on ice. 2 mL of sorting buffer was added and mixed thoroughly. The cell suspension was transferred into a 5 mL flow tube and placed in a magnetic rack for 5 min. This process was repeated twice, and the liquid was poured into a 10 mL EP tube, followed by centrifugation at 300 g, 4°C for 10 min. The pellets obtained were the neutrophil‐like cell [19]. The cells were washed twice with sterile saline injection solution, resuspended, and adjusted to a cell density of 2.5 × 10^7^ cells/mL. On days 3 and 7 after DSS induction, 200 µL of neutrophil suspension was reinfused via the tail vein.

### 2.3 Histopathological Examination of Colonic Tissues

Colonic tissue samples were taken from the rectum of mice and fixed in 10% neutral buffered formalin. They were neutralized in 5% sodium sulfate solution for 36 h, rinsed overnight with running water, dehydrated using a gradient of absolute ethanol, embedded in paraffin, and sectioned. The sections were stained with hematoxylin and eosin (H&E), and examined under an optical microscope to assess the following pathological changes within the intestinal walls. Severity of intestinal inflammation was graded as 1, 2, or 3, representing mild, moderate, and severe inflammation, respectively [20]. Extent of lesions was graded as 1, 2, or 3, indicating lesions in the mucosal layer, mucosa and submucosa, and transmural injury, respectively. Degree of crypt damage was graded as 1, 2, 3, or 4, representing one‐third crypt damage, two‐thirds crypt damage, complete loss of crypts with intact surface epithelium, and complete loss of both crypts and surface epithelium, respectively.

### 2.4 Isolation of Single Cells from the Colonic Mucosal Epithelial Layer and Lamina Propria

The two‐centimeter segments of colon tissues were cut into small pieces and placed in a 50 mL centrifuge tube. Then, 10 mL of RPMI 1640 culture medium containing 1 M EDTA (LABLEAD), 1M DTT (MCE), 50 µg/mL gentamicin (Solarbio), and 5% newborn bovine serum (NBS) were added [21]. The tube was then placed in a tabletop constant‐temperature oscillator at 180 rpm and 37°C, and shaken horizontally for 2 h. Afterward, the supernatant was aspirated and filtered through a 300‐mesh sieve to collect the filtrate. The colon tissues were washed three times with 2 mL of culture medium, and each time the supernatant was aspirated and filtered. All filtrates were combined. The combined filtrates were then centrifuged at 300 g and 4°C for 10 min, and the supernatant was discarded, leaving behind the single cells from the colonic mucosal epithelial layer. For the colon segments subjected to epithelial dissociation, they were placed in 5 mL of RPMI 1640 culture medium containing 60 units/mL type IV collagenase, 50 µg/mL gentamicin, and 5% NBS. The colon segments were then placed in a tabletop constant‐temperature oscillator at 180 rpm and 37°C, and shaken horizontally for 2 h. The suspension was then filtered through a 300‐mesh sieve to obtain the single‐cell suspension of the colonic mucosal lamina propria. Subsequently, the suspension was centrifuged at 300 g and 4°C for 10 min, and the supernatant was discarded, leaving behind the single cells from the colonic mucosal lamina propria.

### 2.5 Flow Cytometry

The prepared single cells from the colonic mucosal epithelial layer and lamina propria were separately incubated with RPMI 1640 medium containing APC anti‐mouse Ly‐6G and FITC anti‐mouse CD11b flow cytometry antibodies, 100 µL each, at 4°C in the dark for 1 h. After the incubation, 100 µL of fixation solution was added and incubated at room temperature in the dark for 30 min [22]. Subsequently, 1 mL of pre‐chilled PBS was added to wash the cells, followed by centrifugation at 2000 rpm and 4°C for 10 min. The supernatant was discarded, and the cells were resuspended in 400 µL of pre‐chilled PBS for analysis by flow cytometry.

### 2.6 Immunofluorescence Staining of Tissues

The mouse colon tissue samples fixed in 10% formalin solution were embedded in paraffin and sectioned. The deparaffinized sections were subjected to antigen retrieval in 95°C citrate buffer (pH 6.0) for 15 min, followed by natural cooling and washing three times with PBS [23]. Sections were then blocked with three percent bovine serum albumin (BSA) solution at room temperature for 1 h. After removing the BSA solution, the sections were washed with PBS and incubated overnight at 4°C in a humid chamber with the primary antibodies. The next day, the sections were incubated with the secondary antibody at room temperature in the dark for 2 h. After removing the secondary antibody, DAPI solution was added, and the sections were incubated at 37°C in the dark for 15 min. Finally, mounting medium was applied, and the slides were sealed.

### 2.7 Cell Migration Assay

Normal human colonic epithelial cells (NCM460 cells) in logarithmic growth phase were seeded at a density of 4×10^5^ cells/mL in a twelve‐well plate and cultured at 37°C with 5% CO2 until reaching 70 to 80% confluence. Subsequently, the cells were starved overnight in serum‐free medium. The culture medium in each well was then replaced with RPMI 1640 medium containing 12% NBS, followed by stimulation with TNF‐α (50 µg/mL) and incubation with RvD5 (1, 3, 10 nm) for 24 h. Cell supernatants were collected thereafter. Neutrophil‐like cells were adjusted to a density of 1 × 10^6^ cells/mL, and 100 µL of cell suspension was seeded in the upper chamber of Transwell inserts. The collected supernatant from NCM460 cell cultures was added to the lower chamber and incubated for 4 h to analyze cell migration through the membrane using cell counting methods [24].

### 2.8 Measurement of cAMP Levels

A total of 200 µL of pre‐chilled PBS containing 100 µm phosphodiesterase inhibitor was added to each well of a 6‐well plate. The cells were harvested using a cell scraper and transferred to a 1.5 mL Eppendorf tube. The cells were subsequently disrupted by sonication for 5 s, followed by interval of 10 s, with the process repeated three times. Following cell disruption, the samples were centrifuged at 12 000 rpm 4°C for 10 min. The supernatant was carefully collected and retained as the sample for analysis. The cAMP content was determined according to the kit (Elabscience) manufacturer's protocol [25].

### 2.9 Analysis of Genes Associated with GPR101

RNA‐seq data was preprocessed by removing low‐quality sequencing reads, normalizing gene expression levels, and ensuring that the data accurately reflected the expression levels of genes within the samples. Using the CIBERSORT algorithm, the relative proportion of neutrophils was estimated through a linear regression model based on gene expression data from mucosal tissues. Based on the median expression level of GPR101 in the mucosal tissue of UC patients, inflammatory bowel disease (IBD) patients from the GSE107499 dataset were categorized into low‐ and high‐expression groups. Differential analysis was conducted to reveal the relationship between changes in GPR101 expression levels and disease status. To further explore the biological functions of GPR101, the GSEA package was utilized for enrichment analysis of GPR101‐related genes to identify biological pathways or processes associated with GPR101 in the gene expression data [26].

### 2.10 Knockdown of GPR101 Expression in Mice

The prepared GPR101‐shRNA or control virus was diluted to 5 × 10^13^ v.p./mL. Excluding the normal group, each mouse received either the overexpression virus or the control virus via intrarectal administration (one‐tenth milliliter per mouse). After 14 days, a DSS‐induced colitis model was established in mice.

### 2.11 Culture and Polarization of Bone Marrow‐derived Macrophages (BMDMs)

After euthanizing C57BL/6 mice by cervical dislocation, their femurs and tibias were swiftly immersed in 75% ethanol for 5 min. Subsequently, under sterile conditions, bone marrow cells were carefully flushed out. These cells were seeded in six‐well plates at a concentration of 5 × 10^6^ cells/mL and cultured for 7 days in the presence of clone 929 cells (L929 cells) conditioned medium. Once mature bone marrow‐derived macrophages (BMDMs) were established, the medium was replaced with DMEM supplemented with 15% FBS, 100 U/mL streptomycin, and 100 U/mL penicillin [27]. IL‐4 and IL‐13 (TargetMol) were added at a final concentration of 10 ng/mL to stimulate polarization toward the M2 phenotype over 48 h period.

### 2.12 Lipid Extraction

The mouse colon tissue sample was centrifuged at 4°C at 2000 g for 10 min, and the supernatant was collected. An equal volume of pre‐chilled chloroform was then added. The sample was sonicated at 25°C for 3 min, followed by centrifugation at 4°C at 4000 rpm for 10 min, and the lower organic phase was extracted. A mixture of chloroform, methanol, and water (2:1:1) was added to the remaining liquid, and the above steps were repeated. The lower organic phases were then combined. The organic phase was evaporated under nitrogen until 95% of the solvent had evaporated, after which the tube walls were rinsed with methanol. After complete solvent evaporation, 100 µL of methanol was added for reconstitution. The sample was centrifuged at 4°C at 2000 g for 5 min, and the supernatant was transferred to a 1.5 mL centrifuge tube. The sample was centrifuged at 4°C at 10 000 g for 10 min, and the supernatant was transferred to a sample vial insert, which was then placed in a clearly labeled sample bottle for UPLC‐MS/MS analysis.

### 2.13 UPLC‐MS/MS Assay

An appropriate amount of berberine hydrochloride was weighed as the internal standard, dissolved in methanol, and diluted to prepare a stock solution with a concentration of 1 mg/mL. Aliquots of the RvD5 solution were accurately pipetted and diluted with methanol to prepare a series of standard solutions at varied concentrations. The peak area ratios of RvD5 relative to berberine hydrochloride were plotted as a function of standard solution concentrations. Linear regression was subsequently performed using the method of least squares to derive the linear equation. Concentrations yielding signal‐to‐noise ratios of three and ten for chromatographic peaks relative to the baseline were defined as the limits of detection and quantification, respectively [28].

### 2.14 Myeloperoxidase (MPO) Activity Assay

MPO activity was measured using a commercial assay kit (Nanjing Jiancheng Bioengineering Institute). Tissue samples were homogenized, and the supernatant was collected and loaded into 96‐well plates, with parallel background control wells included. The reaction mixture was then added, and samples were incubated at 37°C for 30 min. Absorbance was measured at 460 nm, and MPO activity was expressed as units per gram of wet tissue weight (U/g).

### 2.15 Statistical Analysis

All data are presented as means ± standard errors of the means (S.E.M.). Statistical differences between groups were analyzed using one‐way ANOVA and two‐way ANOVA in GraphPad Prism 9 software. A *p*‐value less than 0.05 was considered statistically significant.

## 3 Results

### 3.1 RvD5 Attenuates DSS‐Induced Colitis in Mice and Neutrophil Infiltration into Colonic Mucosal Epithelium

**FIGURE 1:**
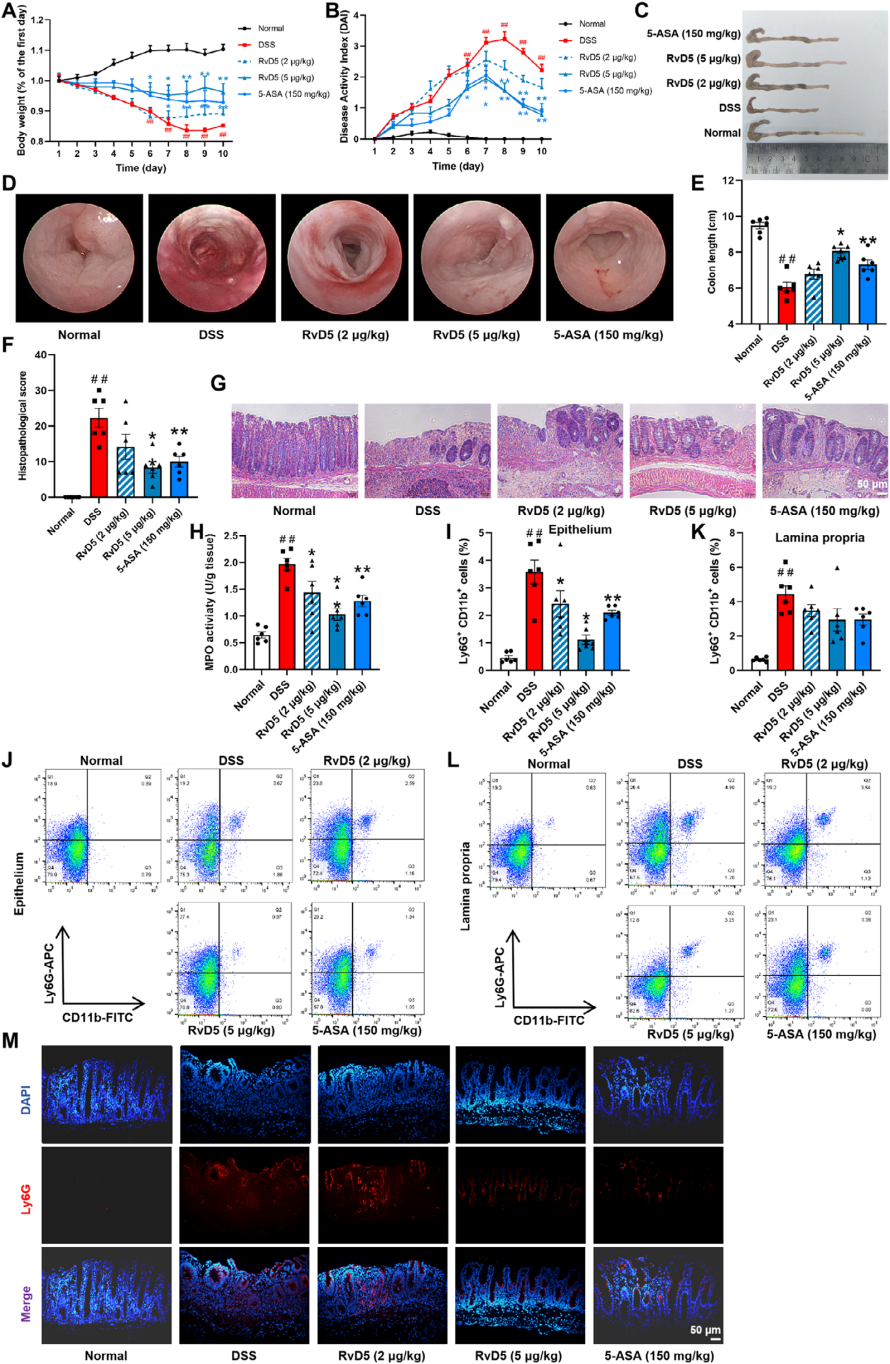
Effect of RvD5 on DSS‐induced colitis in mice and neutrophil infiltration in the colon. Colitis was induced in C57BL/6 mice by administering 2.5% DSS in drinking water for 7 consecutive days, followed by normal drinking water for 3 consecutive days. RvD5 (2, 5 µg/kg) was intraperitoneally injected and 5‐ASA (150 mg/kg) was orally administered for 10 consecutive days. (A) Percentage change in body weight. (B) DAI score. (C, E) Colon length. (D) Representative images of endoscopy. (F, G) Pathological score was assessed by H&E‐stained (scale bar, 50 µm). (H) MPO activity in colons was detected by commercial kits. (I, J) Percentage of Ly6G^+^CD11b^+^ cells in the mononuclear cells of epithelium from mouse colon tissues was detected by flow cytometry. (K, L) Percentage of Ly6G^+^CD11b^+^ cells in the mononuclear cells of lamina propria from mouse colon tissues was detected by flow cytometry. (M) Representative immunofluorescent stained images of Ly6G (red) in colon tissues (scale bar, 50 µm). Data are presented as the mean ± S.E.M. of six mice per group. ^##^ *P* < 0.01 vs the normal group; ^*^ *P* < 0.05, ^**^ *P* < 0.01 vs the DSS group.

An experimental colitis was established in mice using 2.5% DSS. The treatment groups received either intraperitoneal injection of RvD5 (2, 5 µg/kg) or oral administration of the positive control drug 5‐ASA (150 mg/kg) for 10 consecutive days. The colitis symptoms of mice, including body weight decrease, diarrhea severity, and rectal bleeding, were observed, and the disease activity index (DAI) was calculated. Both RvD5 (5 µg/kg) and 5‐ASA (150 mg/kg) significantly alleviated weight loss (Figure 1A) and downregulated DAI scores of colitis mice (Figure 1B). The effect of RvD5 (5 µg/kg) was superior to that of 5‐ASA (150 mg/kg). The colon mucosa of DSS‐treated mice exhibited prominent congestion and edema, with blurred colonic folds, widespread erosion and ulceration, and increased mucus secretion. Compared to the DSS group, RvD5 (5 µg/kg) and 5‐ASA (150 mg/kg) treatments significantly ameliorated colonic mucosal damage in colitis mice (Figure 1C,D). Both interventions also effectively suppressed the shortening of the colon induced by DSS (Figure 1E). Moreover, both treatments significantly ameliorated histopathological changes in colitis mice (Figure 1F,G). These results suggest that exogenous supplement of RvD5 can effectively ameliorate DSS‐induced colitis in mice.

The extensive infiltration of neutrophils disrupts the integrity of the epithelial barrier and concurrently releases pro‐inflammatory mediators, which further recruit and activate other immune cells to exacerbate mucosal damage and intestinal inflammation. RvD5 (2, 5 µg/kg) and 5‐ASA (150 mg/kg) treatments significantly reduced the MPO activity in colonic tissue of colitis mice (Figure 1H). To delve deeper into the impact of RvD5 on neutrophil infiltration in the colonic mucosa, single‐cell suspensions of colonic mucosal epithelial layer and lamina propria layer were prepared. Compared to the DSS group, RvD5 (2, 5 µg/kg) and 5‐ASA (150 mg/kg) significantly decreased the proportion of neutrophils in the colonic mucosal epithelium. Notably, the inhibitory effect of RvD5 (5 µg/kg) on neutrophil infiltration in the colonic mucosal epithelium was greater than that of 5‐ASA (150 mg/kg) (Figure 1I,J). In contrast, RvD5 (2, 5 µg/kg) and 5‐ASA (150 mg/kg) treatments were lack of significant effect on the increased proportion of neutrophils in the colonic mucosal lamina propria layer of colitis mice (Figure 1K,L). Immunofluorescence assay results also demonstrated a significant increase in the number of neutrophils in both the colonic mucosal epithelial and lamina propria layers of DSS group mice. Compared to the DSS group mice, RvD5 (2, 5 µg/kg) and 5‐ASA (150 mg/kg) significantly reduced the number of neutrophils in the colonic mucosal epithelium (Figure 1M). These findings suggest that RvD5 effectively inhibits neutrophil infiltration in the colonic mucosal epithelium of mice with DSS‐induced colitis.

### 3.2 Importance of Preventing Neutrophil Infiltration in the Anti‐Colitis Effect of RvD5

**FIGURE 2:**
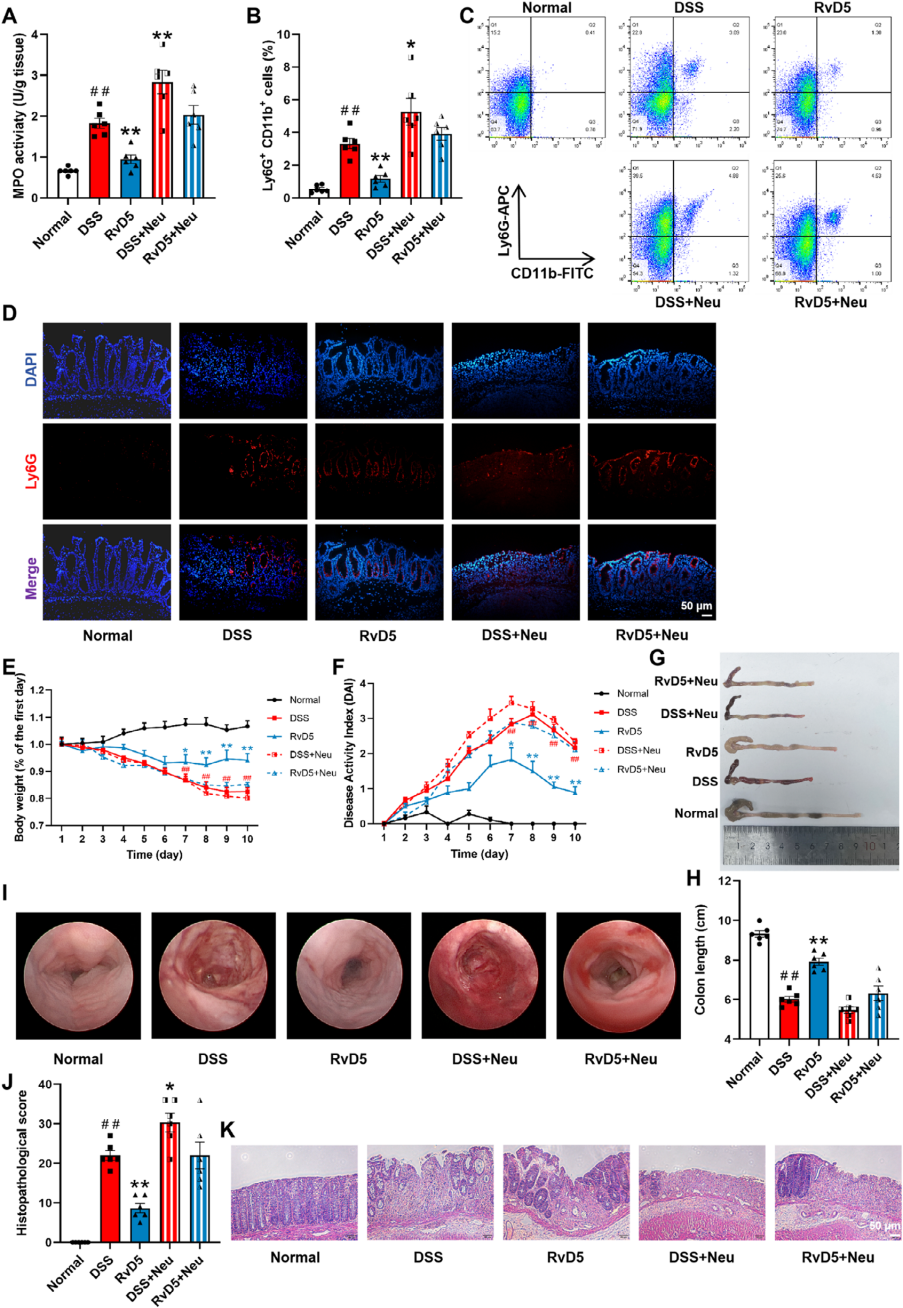
Effect of adoptive transfer of neutrophils (Neu) on the alleviation of RvD5 against mouse colitis. On the third and seventh days after induction of colitis by DSS, adoptive transfer of bone marrow‐derived neutrophils isolated from syngeneic mice was conducted. (A) MPO activity in colons was detected by commercial kits. (B, C) Percentage of Ly6G^+^CD11b^+^ cells in the mononuclear cells of epithelium from mouse colon tissues was detected by flow cytometry. (D) Representative immunofluorescent stained images of Ly6G (red) in colon tissues (scale bar, 50 µm). (E) Percentage change in body weight. (F) DAI score. (G, H) Colon length. (I) Representative images of endoscopy. (J, K) Pathological score was assessed by H&E stain (scale bar, 50 µm). Data are presented as the mean ± S.E.M. of six mice per group. ^##^ *P* < 0.01 vs the normal group; ^*^ *P* < 0.05, ^**^ *P* < 0.01 vs the DSS group.

In mice with DSS‐induced colitis, adoptive transfer of neutrophils significantly diminished the effect of RvD5 to reduce MPO activity in colonic tissues (Figure 2A). It also markedly weakened the ability of RvD5 to decrease the proportion and number of neutrophils in the colonic mucosal epithelium (Figure 2B–D). Compared to the normal group, DSS group mice exhibited colitis symptoms such as weight loss, diarrhea, and hematochezia, with a significant increase in DAI score. Adoptive transfer of neutrophils elevated the DAI score in colitis mice, while significantly attenuated the ameliorative effect of RvD5 on the colitis symptoms mentioned above (Figure 2E,F). It also exacerbated colonic shortening in colitis mice and diminished the alleviating effect of RvD5 on the colonic shortening (Figure 2G,H). Furthermore, adoptive transfer of neutrophils significantly weakened the effect of RvD5 to improve colonic mucosal damage and histopathological changes in colitis mice (Figure 2I–K). These results suggest that adoptive transfer of neutrophils substantially attenuates the ability of RvD5 (5 µg/kg) to suppress neutrophil infiltration and tissue damage in the colonic mucosal epithelium of mice with colitis.

### 3.3 RvD5 Inhibits Neutrophil Chemotaxis Toward Colonic Epithelial Cells by Downregulating CXCL8 Expression

The findings that RvD5 specifically inhibited neutrophil infiltration in the colonic mucosal epithelial layer rather than the lamina propria suggest that it exerts anti‐colitis action by downregulating the expression of chemokines in epithelial cells. In the primary colonic epithelial cells isolated from mice with DSS‐induced colitis, quantitative PCR (Q‐PCR) and ELISA were employed to assess the expression of neutrophil‐related chemokines. The results showed that the mRNA expression of CXCL1 (homologous to human CXCL8), CXCL7, CXCL10, and CCL20 in primary colonic epithelial cells from DSS‐treated mice was markedly increased. Compared to DSS‐treated group, both RvD5 (2, 5 µg/kg) and 5‐ASA (150 mg/kg) markedly downregulated the mRNA expression of CXCL1, but not other chemokines, in primary colonic epithelial cells of colitis mice (Figure S1A–D). Notably, the inhibitory effect of RvD5 (5 µg/kg) on CXCL1 mRNA expression was greater than that of 5‐ASA (150 mg/kg). The protein level of CXCL1 in colonic epithelial cells was also markedly increased in mice with DSS‐induced colitis compared to mice in the normal group, and both RvD5 (2, 5 µg/kg) and 5‐ASA (150 mg/kg) significantly downregulated CXCL1 protein expression in primary colonic epithelial cells of colitis mice (Figure S1E). These results suggest that the inhibition of RvD5 on neutrophil recruitment in the colonic mucosal epithelial layer of colitis mice is likely due to the downregulation of CXCL1 expression in colonic epithelial cells.

**FIGURE 3:**
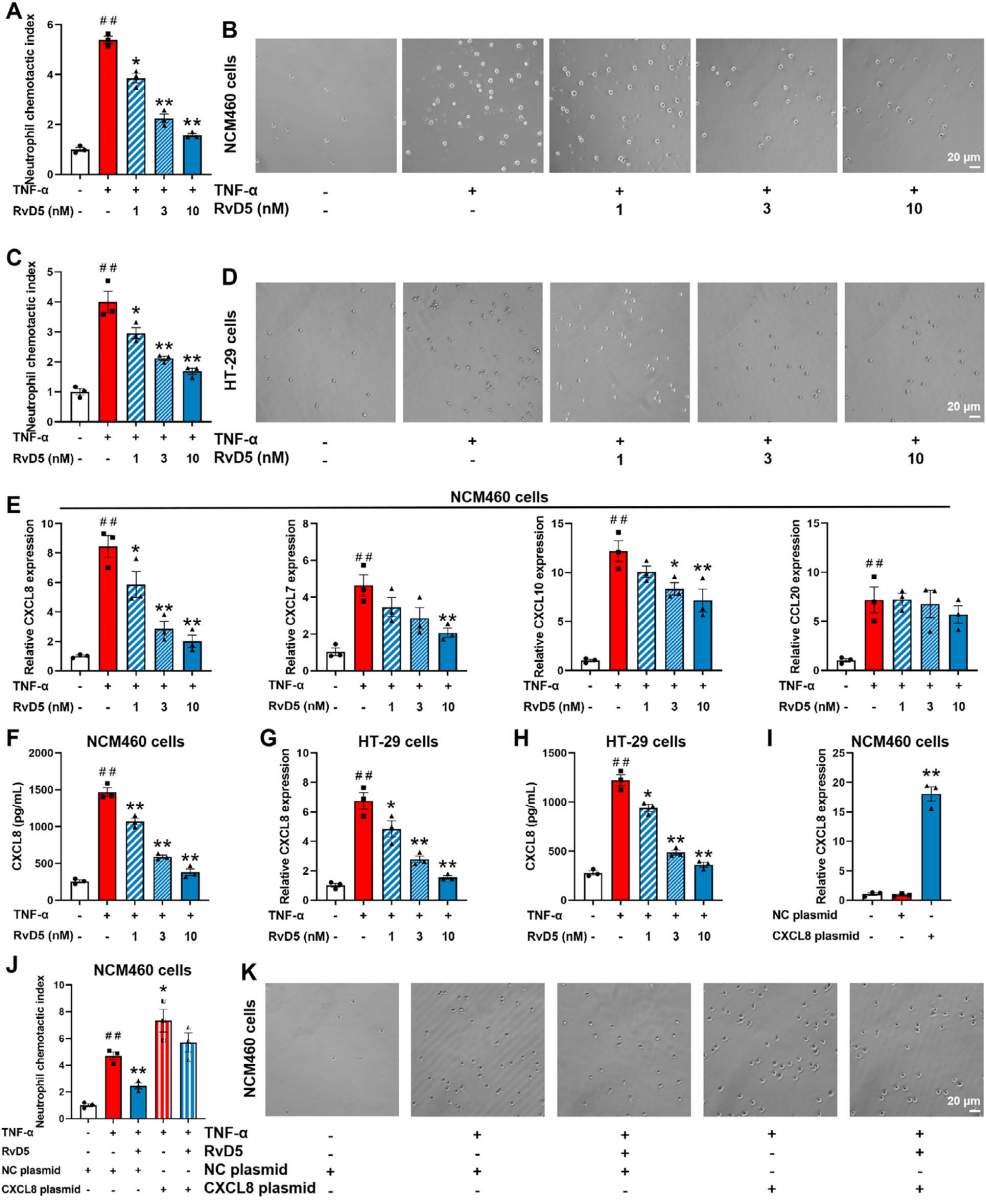
Effect of RvD5 on the expression of chemokines in colonic epithelial cells. Colonic epithelial cells (NCM460 cells and HT‐29 cells) were pre‐stimulated with TNF‐α (50 ng/mL) and subsequently treated with varying concentrations of RvD5 (1, 3, 10 nm) for 24 h prior to collecting the cell supernatants. Neutrophil‐like cells were seeded in the upper chamber of Transwell inserts, and the collected supernatants from colonic epithelial cell cultures were added to the lower chamber of co‐incubation for 4 h. Cell counting analysis was conducted on the cells that migrated to the lower chamber. (A, B) The chemotactic index of neutrophils toward NCM460 cells was detected by Transwell assay. (C, D) The chemotactic index of neutrophils toward HT‐29 cells was detected by Transwell assay. (E) The mRNA expression of CXCL8, CXCL7, CXCL10, and CCL20 in NCM460 cells was detected by Q‐PCR. (F) The protein expression of CXCL8 in NCM460 cells was detected by western blotting. (G) The mRNA expression of CXCL8 in HT‐29 cells was detected by Q‐PCR. (H) The protein expression of CXCL8 in HT‐29 cells was detected by western blotting. (I) The overexpression efficiency was detected by Q‐PCR detection. (J, K) The chemotactic index of neutrophils toward NCM460 cells was detected by Transwell assay (scale bar, 20 µm). Data are presented as the mean ± S.E.M from three independent experiments. ^##^ *P* < 0.01 vs the control group; ^*^ *P* < 0.05, ^**^ *P* < 0.01 vs the treatment group.

To investigate the effect of RvD5 on neutrophil chemotaxis, a transwell cell migration assay was conducted. Under TNF‐α (50 ng/ml) stimulation, co‐culture with conditioned media from NCM460 cells significantly increased the proportion of neutrophil‐like cells crossing the transwell chamber. In contrast, conditioned media from NCM460 cells treated with RvD5 (3, 10 nm) markedly suppressed the chemotactic effect of neutrophil‐like cells (Figure 3A,B). Similarly, conditioned media from HT‐29 cells treated with RvD5 (3, 10 nm) significantly inhibited neutrophil‐like cells chemotaxis (Figure 3C,D). TNF‐α (50 ng/ml) stimulation significantly increased the mRNA expression of CXCL8, CXCL7, CXCL10, and CCL20 in NCM460 cells. Treatment with RvD5 (3, 10 nm) selectively downregulated mRNA expression of CXCL8 in NCM460 cells (Figure 3E). Moreover, RvD5 (1, 3, 10 nm) significantly reduced protein expression of CXCL8 in NCM460 cells (Figure 3F). Similarly, RvD5 (3, 10 nm) markedly decreased both mRNA and protein expression of CXCL8 in HT‐29 cells (Figure 3G,H). To explore whether RvD5 inhibits neutrophil chemotaxis toward colonic epithelial cells by downregulating CXCL8 expression, we constructed a CXCL8 overexpression plasmid and transfected it into NCM460 cells. CXCL8 overexpression significantly increased mRNA expression of CXCL8 in NCM460 cells, confirming successful overexpression (Figure 3I). CXCL8 overexpression elevated the neutrophil‐like cells chemotaxis index in conditioned media from NCM460 cells and attenuated the inhibitory effect of RvD5 (3 nm) on the chemotaxis of neutrophil‐like cells toward colonic epithelial cells (Figure 3J,K). These results indicate that RvD5 suppresses neutrophil chemotaxis toward colonic epithelial cells by selectively downregulating the expression of CXCL8.

### 3.4 RvD5 Downregulates CXCL8 Expression and Neutrophil Chemotaxis in Colonic Epithelial Cells Through Activation of GPR101

**FIGURE 4:**
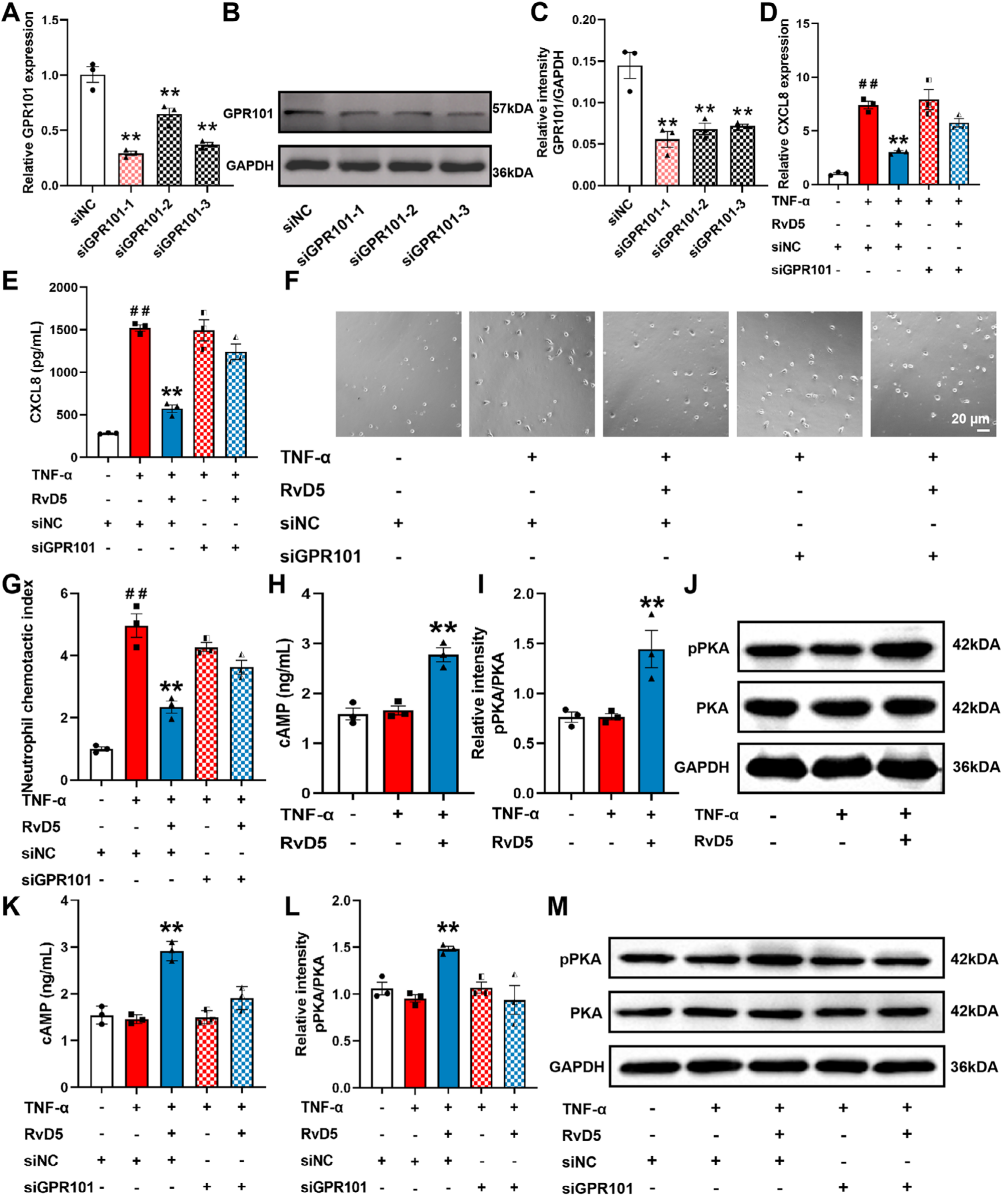
RvD5 downregulates CXCL8 expression in colonic epithelial cells and neutrophil chemotaxis in the GPR101‐dependent manner. NCM460 cells were transfected with NC siRNA or GPR101 siRNA (siR‐GPR101) for 72 h. (A) The knockdown efficiency was detected by Q‐PCR. (B, C) The protein expression of GPR101 was detected by western blotting. (D) The mRNA expression of CXCL8 in NCM460 cells was detected by Q‐PCR. (E) The protein expression of CXCL8 in NCM460 cells was detected by western blotting. (F, G) The chemotactic index of neutrophils toward NCM460 cells was detected by Transwell assay (scale bar, 20 µm). NCM460 cells were pre‐stimulated with TNF‐α (50 ng/mL) followed by treatment of RvD5 (3 nm) for 2 h before the extraction of intracellular cAMP and proteins. (H) Intracellular cAMP levels in colonic epithelial cells were detected by ELISA kit. (I, J) The protein expression of PKA and pPKA in colonic epithelial cells was detected by western blotting. (K) Intracellular cAMP levels in colonic epithelial cells were detected by ELISA kit. (L, M) The protein expression of PKA and pPKA in colonic epithelial cells was detected by western blotting. Data are presented as the mean ± S.E.M from three independent experiments. ^##^ *P* < 0.01 vs the control group; ^*^ *P* < 0.05, ^**^ *P* < 0.01 vs the treatment group.

RVD5 exerts biological effects primarily through activation of the G protein‐coupled receptors GPR32 and GPR101. To investigate which receptor mediates the inhibitory effect of RvD5 on CXCL8 expression and neutrophil chemotaxis, we used siRNA to knock down GPR32 and GPR101 expression in colonic epithelial cells, respectively. After siRNA‐mediated GPR32 knockdown in NCM460 cells, RvD5 (3 nm) still significantly decreased CXCL8 expression at mRNA and protein levels and inhibited neutrophil‐like cell chemotaxis toward NCM460 cells (Figure S2A–G). In contrast, knocking down GPR101 in NCM460 cells significantly weakened the ability of RvD5 (3 nm) to reduce CXCL8 expression at mRNA and protein levels (Figure 4A–E), and diminished the inhibitory effect of RvD5 (3 nm) on neutrophil‐like cell chemotaxis toward NCM460 cells (Figure 4F,G). These results indicate that GPR101 mediates the inhibition of RvD5 against CXCL8 expression in colonic epithelial cells and neutrophil chemotaxis toward these cells.

To ascertain the activation of GPR101 by RvD5 in colonic epithelial cells, we measured intracellular cAMP levels using an ELISA kit and PKA phosphorylation levels by Western blotting. RvD5 (3 nm) significantly increased intracellular cAMP levels and promoted PKA phosphorylation in NCM460 cells (Figure 4H–J). Knocking down GPR101 attenuated RvD5's ability to elevate intracellular cAMP levels and promote PKA phosphorylation in colonic epithelial cells (Figure 4K–M). These findings suggest that RvD5 downregulates CXCL8 expression in colonic epithelial cells and neutrophil chemotaxis through activation of GPR101 and its downstream signaling pathways.

### 3.5 Activation of GPR101 Downregulates the Expression of CXCL8 via Transcription Factor STAT1

**FIGURE 5:**
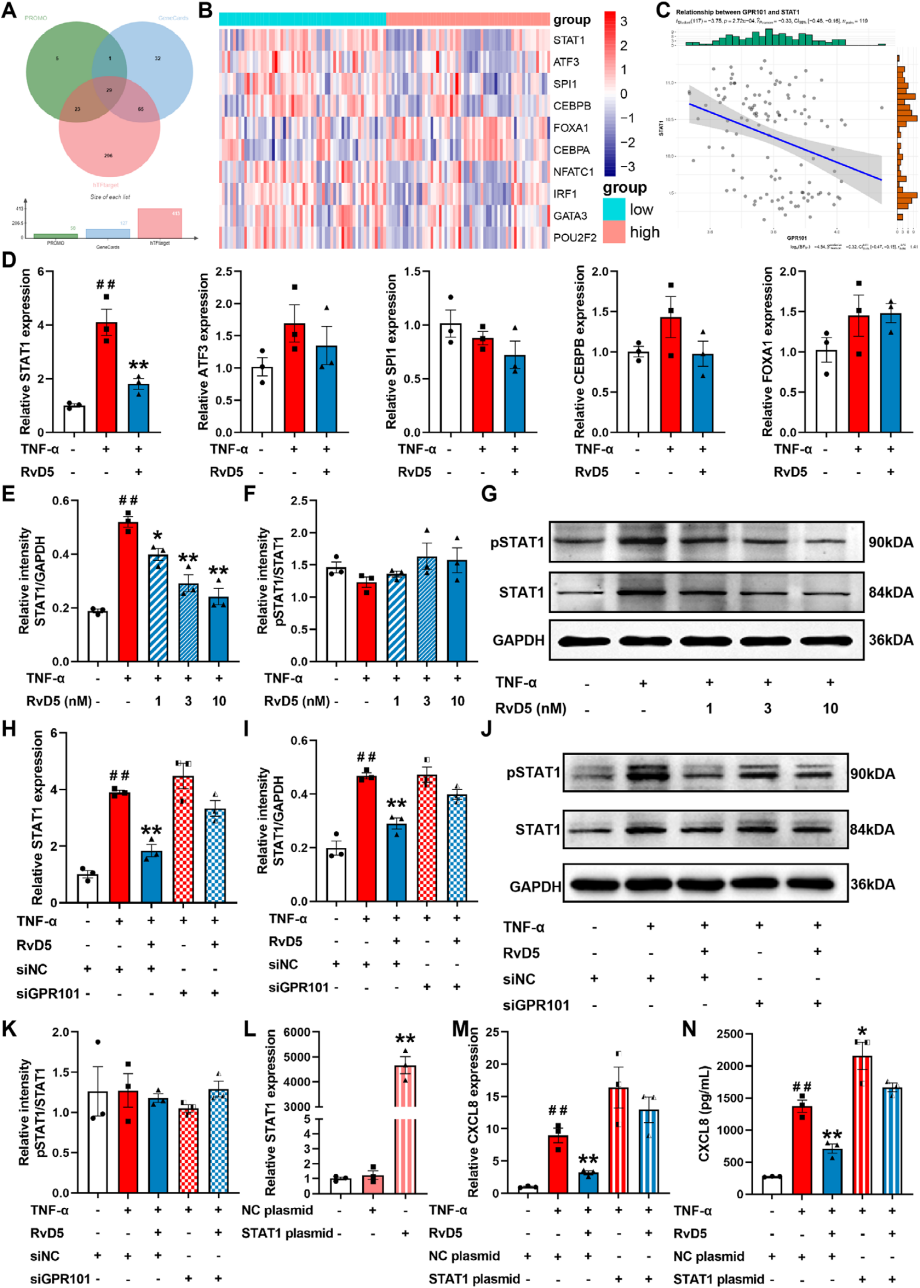
The signaling pathway through which RvD5 activates GPR101 to downregulate CXCL8 expression. (A) Schematic representation of the intersection of transcription factors predicted by GeneCards, PROMO, and humanTFDB databases. (B) Heatmap illustrating the expression of the top 10 CXCL8‐related transcription factors with the smallest P‐values in mucosal tissue of UC patients. (C) Correlation analysis of CXCL8 and STAT1 mRNA expression. NCM460 cells were pre‐stimulated with TNF‐α (50 ng/mL) and then treated with RvD5 for 24 h. (D) The mRNA expression of STAT1, ATF3, SPI1, CEBPB, and FOXA1 in NCM460 cells was detected by Q‐PCR. (E–G) The protein expression of STAT1 and pSTAT1 was detected by western blotting. NCM460 cells were transfected with NC siRNA or GPR101 siRNA, pre‐stimulated with TNF‐α (50 ng/mL), and then treated with RvD5 (3 nm) for 24 h. (H) The mRNA expression of STAT1 in NCM460 cells was detected by Q‐PCR. (I‐K) The protein expression of STAT1 and pSTAT1 in NCM460 cells was detected by western blotting. (L) The mRNA expression of STAT1 in NCM460 cells transfected with pcDNA3.1‐STAT1 plasmid or empty vector was detected using Q‐PCR. (M) The mRNA expression of STAT1 in NCM460 cells was detected by Q‐PCR. (N) The protein expression of STAT1 and pSTAT1 in NCM460 cells was detected by western blotting. Data are presented as the mean ± S.E.M from three independent experiments. ^##^ *P* < 0.01 vs the control group; ^*^ *P* < 0.05, ^**^ *P* < 0.01 vs the treatment group.

Bioinformatics analysis of RNA‐seq data from mucosal tissues of UC patients in the GSE107499 dataset revealed that the expression level of GPR101 significantly decreased at the lesion sites, which correlated negatively with the extent of neutrophil infiltration (Figure S3A–D). To elucidate the specific mechanism by which RvD5 inhibits CXCL8 expression through GPR101 activation, we predicted potential transcription factors using several databases. GeneCards database identified 127 transcription factors, Human TFDB predicted 413, and PROMO identified 58, with 28 overlapping transcription factors across all three databases (Figure 5A). UC patients were grouped based on GPR101 expression levels, and a differential analysis of these transcription factors between the groups was conducted. We extracted expression data for the top 10 transcription factors with the smallest *p*‐values and constructed a heatmap (Figure 5B). A significant negative correlation was observed between GPR101 expression and STAT1 level in colonic mucosal tissues of UC patients (Figure 5C). To further investigate the key transcription factors, we used Q‐PCR to measure the expression of the top 5 transcription factors identified. TNF‐α (50 ng/ml) stimulation significantly increased the mRNA expression of STAT1 in NCM460 cells, while slightly increased the mRNA expression of ATF3, CEBPB, and FOXA1, and slightly decreased the mRNA expression of SPI1. RvD5 (3 nm) significantly downregulated the mRNA expression of STAT1 in NCM460 cells treated with TNF‐α (Figure 5D). Western blotting analysis showed that RvD5 (3 nm) had no significant effect on the protein expression of ATF3, SPI1, CEBPB, and FOXA1 (Figure S4A–E). Under TNF‐α stimulation, STAT1 and pSTAT1 protein levels significantly increased in NCM460 cells. RvD5 (3 nm) significantly reduced STAT1 and pSTAT1 protein expression levels in these cells, while the pSTAT1/STAT1 ratio was unaffected, suggesting that RvD5 primarily suppresses STAT1 expression rather than its phosphorylation activity. (Figure 5E–G).

Similar results were obtained in HT‐29 cells (Figure S5A–D). Knockdown of GPR101 using siRNA in NCM460 cells significantly weakened the inhibitory effect of RvD5 (3 nm) on STAT1 expression (Figure 5H–K). Overexpression of STAT1 via plasmid transfection in NCM460 cells significantly attenuated the inhibitory effect of RvD5 (3 nm) on CXCL8 expression (Figure 5L–N). Ruxolitinib, a pSTAT1 inhibitor, markedly reduced CXCL8 expression in NCM460 cells. Furthermore, in the presence of Ruxolitinib (3 nm), RvD5 (3 nm) failed to significantly further suppress CXCL8 expression (Figure S6A,B). These findings demonstrate that RvD5 downregulates CXCL8 expression through GPR101‐mediated suppression of STAT1 signaling.

### 3.6 RvD5 activates GPR101 to Downregulate CXCL8 Expression in Colonic Epithelial Cells of Colitis Mice, Thereby Inhibiting Neutrophil Infiltration in Mucosal Epithelium and Alleviating Colitis

To examine the in vivo activation of GPR101 in colonic epithelial cells by RvD5, we established a mouse colitis model using 2.5% DSS. RvD5 was administered via intraperitoneal injection, and the primary colonic epithelial cells were isolated. Intracellular cAMP levels were quantified using an ELISA kit, and PKA phosphorylation was assessed via Western blotting. Q‐PCR analysis showed that GPR101 expression was significantly decreased in colon tissues of DSS‐induced colitis mice compared with the normal group, consistent with the trend observed in ulcerative colitis patient samples (Figure S7A). Treatment with RvD5 (5 µg/kg) slightly increased GPR101 expression in the colonic tissues of DSS‐induced colitis mice; however, the change was not statistically significant. In contrast, RvD5 (5 µg/kg) significantly increased intracellular cAMP levels and promoted PKA phosphorylation in colonic epithelial cells derived from DSS‐induced colitis mice (Figure S7B–D). These results indicate that RvD5 primarily restores the functional activity of GPR101, rather than upregulating its expression, in the colonic tissues of DSS‐induced colitis mice. To verify if RvD5 downregulates CXCL8 expression in colonic epithelial cells of colitis mice through GPR101 activation, thereby inhibiting mucosal neutrophil infiltration and alleviating colitis, we used an AAV virus to interfere with mouse GPR101 expression (Figure S7E). Knockdown of GPR101 slightly upregulated STAT1 expression in primary colonic epithelial cells of DSS‐induced colitis mice, while significantly weakened the inhibitory effect of RvD5 (5 µg/kg) on STAT1 expression (Figure S57F–I). Knockdown of GPR101 significantly upregulated CXCL8 expression in primary colonic epithelial cells derived from DSS‐induced colitis mice and markedly attenuated the inhibitory effect of RvD5 (5 µg/kg) on CXCL8 expression (Figure S7J,K). These results indicate that RvD5 activates GPR101 to downregulate STAT1 expression in colonic epithelial cells of DSS‐induced colitis mice, thereby inhibiting CXCL8 expression.

**FIGURE 6:**
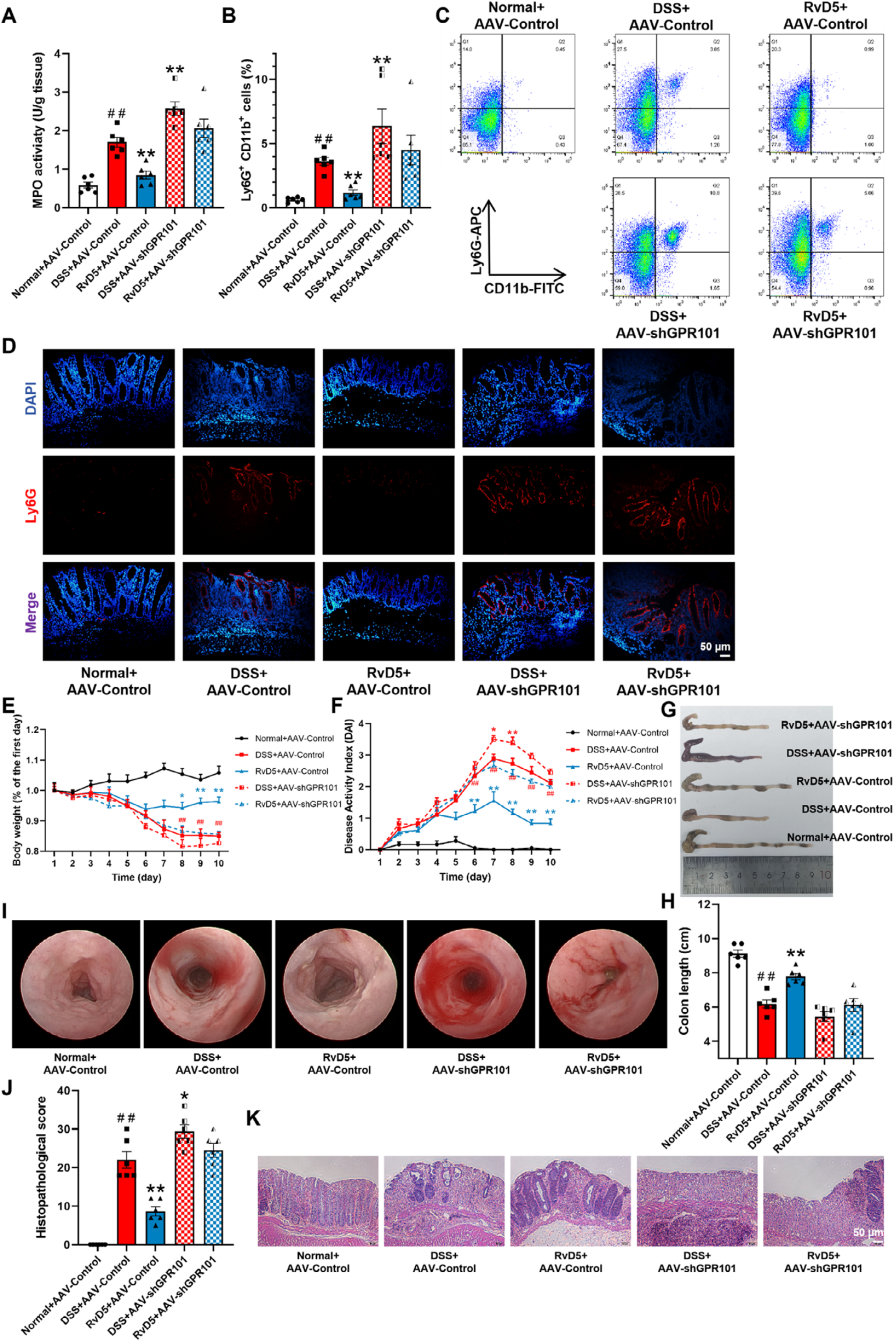
The GPR101‐dependent attenuation of neutrophil infiltration in the colonic mucosal epithelium and colitis in mice by RvD5. Colitis was induced in female C57BL/6 mice by administering 2.5% DSS in drinking water for 7 consecutive days, followed by normal water for 3 consecutive days. RvD5 was continuously administered via intraperitoneal injection at a dosage of 5 µg/kg for 10 consecutive days. Two weeks before induction of colitis by DSS, mice were intrarectally administered the AAV‐shGPR101 virus to interfere with GPR101 expression in colon tissues. (A) MPO activity in colons was detected by commercial kits. (B, C) Percentage of Ly6G^+^CD11b^+^ cells in the mononuclear cells of epithelium from mouse colon tissues was detected by flow cytometry. (D) Representative immunofluorescence staining images of Ly6G (red) in colonic tissues of mice (scale bar, 50 µm). (E) Percentage change in body weight. (F) DAI score. (G, H) Colon length. (I) Representative images of endoscopy. (J, K) Pathological score was assessed by H&E‐stained (scale bar, 50 µm). Data are presented as the mean ± S.E.M. of six mice per group. ^##^ *P* < 0.01 vs the normal group; ^*^ *P* < 0.05, ^**^ *P* < 0.01 vs the DSS group.

Compared to the DSS group, GPR101 knockdown significantly increased MPO activity in colonic tissue of colitis mice, and markedly attenuated RvD5 (5 µg/kg)‐caused downregulation of MPO activity in colonic tissues (Figure 6A). GPR101 knockdown significantly increased the proportion of neutrophils in the colonic mucosal epithelium compared to the DSS group and markedly attenuated RvD5's reduction of neutrophil proportion (Figure 6B,C). GPR101 knockdown significantly increased the number of neutrophils in the colonic mucosa compared to the DSS group and markedly attenuated RvD5's reduction of neutrophil count (Figure 6D). GPR101 knockdown significantly increased the DAI scores in colitis mice compared to the DSS group and markedly attenuated the alleviation of RvD5 on colitis symptoms such as weight loss, diarrhea, and rectal bleeding (Figure 6E,F). Compared to the DSS group, GPR101 knockdown slightly aggravated colonic shortening in colitis mice but significantly attenuated RvD5's effect on alleviating colonic shortening (Figure 6G,H). Compared to the DSS group, mice with GPR101 knockdown exhibited severe colonic mucosal congestion and edema, nearly disappearing colonic folds, with connected ulcers and erosions. After combined treatment with RvD5, GPR101 knockdown significantly weakened the effect of RvD5 on improving colonic mucosal damage in colitis mice (Figure 6I). Compared to the DSS group, GPR101 knockdown significantly exacerbated colonic tissue pathological damage in mice and markedly attenuated the improvement of RvD5 on colonic tissue pathological damage (Figure 6J,K).

### 3.7 5‐LOX Allosteric Inhibitor Epimedin A1 Promotes the Biosynthesis of RvD5 in M2 Macrophage

We observed that RvD5 markedly downregulated CXCL8 expression in colonic epithelial cells and effectively suppressed neutrophil infiltration in the mucosal epithelium, suggesting its potential as a candidate therapeutic agent for UC. As SPMs are rapidly metabolized and inactivated in vivo, therapeutic strategies aimed at promoting its endogenous biosynthesis may offer greater translational promise than direct supplementation of exogenous RvD5 [29]. Recent studies have shown that allosteric inhibitors of 5‐LOX possess the potential to enhance RvD5 biosynthesis [30, 31]. Thus, we conducted molecular docking simulations based on the binding site of the 5‐lipoxygenase (5‐LOX) allosteric inhibitor 3‐acetyl‐11‐keto‐β‐boswellic acid to investigate the interaction between 2560 active components occurring in traditional Chinese medicines and the RvD5 biosynthetic enzyme 5‐LOX. Subsequently, 10 potential 5‐LOX allosteric inhibitors were screened out (Table S1).

**FIGURE 7:**
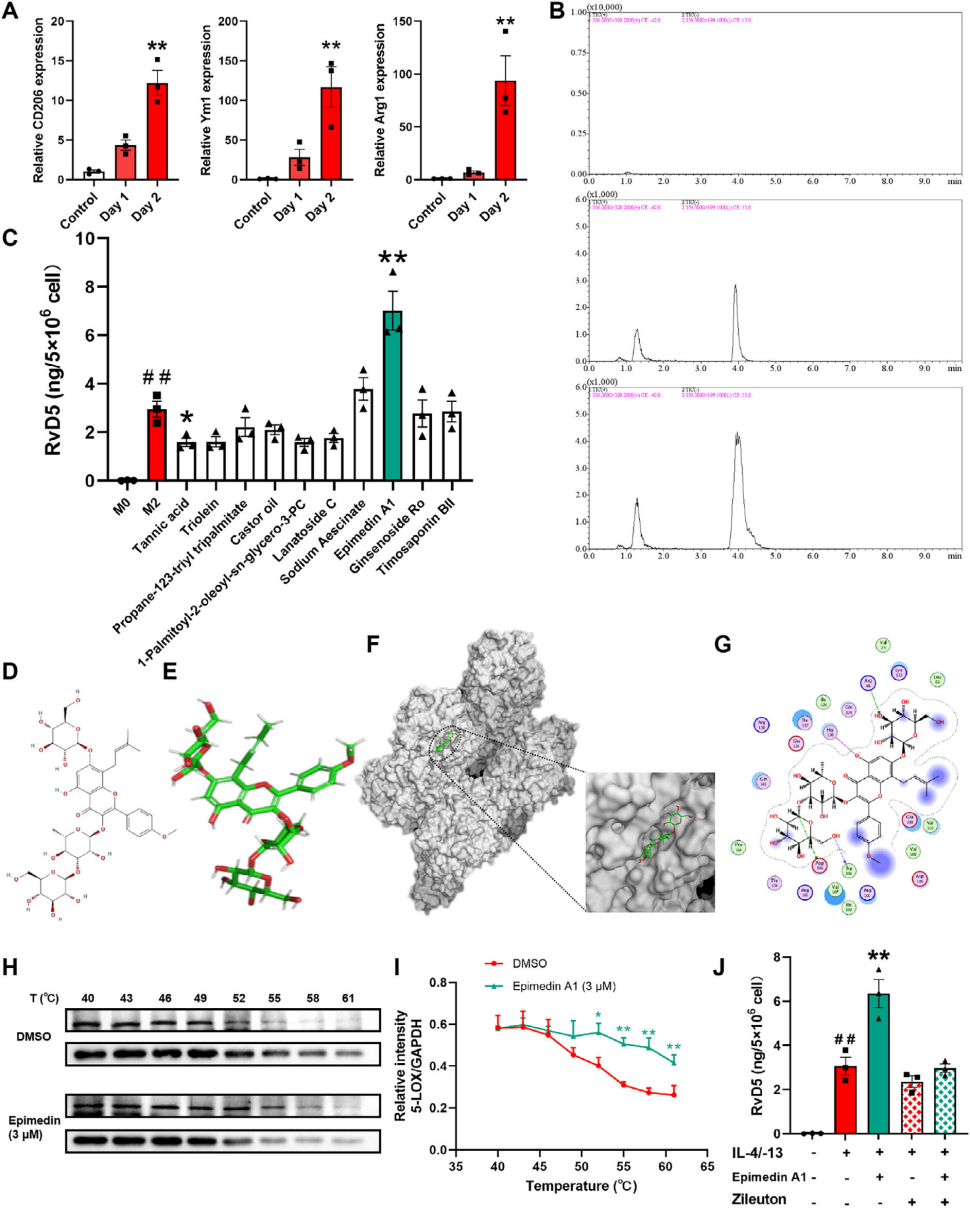
Promotion of RvD5 biosynthesis in M2 macrophage by epimedin A1. An in vitro M2 macrophage polarization system was established using IL‐4 (10 ng/mL) and IL‐13 (10 ng/mL) as induction agents, with subsequent detection of mRNA expression of M2 macrophage markers using Q‐PCR. (A) On 24 h and 48 h after induction of IL‐4 and IL‐13, the mRNA expression of M2 macrophage markers CD206, Ym1, and Arg1 was detected by Q‐PCR, respectively. (B) Chromatographic profile of blank samples, standard solutions, and sample solutions. The first peak corresponds to berberine hydrochloride, and the second peak corresponds to RvD5. (C) M2 macrophages were treated with candidate compounds at a concentration of 3 µm for 2 h, and the content of RvD5 in the cell culture supernatant was detected using UPLC‐MS/MS technology. (D, E) 2D and 3D structure diagrams of epimedin A1. (F) Surface rendering depicts the binding of epimedin A1 with 5‐LOX, where the compound (depicted in green) is positioned within the crevice between the catalytic domain and the membrane‐binding domain. (G) Interaction between epimedin A1 and the docking pocket of 5‐LOX is visualized. (H, I) Protein stability assessment in M2 macrophages following incubation with 3 µm epimedin A1 for 2 h was assessed by CETSA. (J) RvD5 levels secreted by M2 macrophages co‐treated with the 5‐LOX inhibitor Zileuton (1 µm) was performed using UPLC‐MS/MS. Data are presented as the mean ± S.E.M from three independent experiments. ^##^ *P* < 0.01 vs the control group; ^*^ *P* < 0.05, ^**^ *P* < 0.01 vs the treatment group.

Given that M2 macrophages serve as the principal producers of RvD5, [31, 32] we subsequently polarized BMDMs toward an M2 phenotype in vitro. This approach was used to assess whether the candidate compounds could stimulate endogenous RvD5 biosynthesis in M2 macrophages. After induction with IL‐4 and IL‐13 for 48 h, the mRNA expression of M2 macrophage markers CD206, Ym1, and Arg1 significantly increased compared to the control group (Figure 7A), indicating successful polarization of M2 macrophages. M2 macrophages were treated separately with 3 µm of each candidate compound for 2 h, and RvD5 content in the supernatant was detected using UPLC‐MS/MS technology. Blank samples showed minimal baseline noise, indicating no interference in the determination of samples. The retention time of RvD5 was 4.174 min, and the retention time of the internal standard (berberine hydrochloride) was 1.295 min, indicating the reliability of the detection method (Figure 7B). Among the 10 compounds, epimedin A1 (3 µm) was shown to significantly increase the level of RvD5 in M2 macrophages, suggesting that it is a promoter of RvD5 biosynthesis (Figure 7C).

To determine if epimedin A1 directly interacts with 5‐LOX, we used molecular docking to simulate its binding with 5‐LOX. Figure 7D,E shows the 2D and 3D structures of epimedin A1 using MOE for semi‐flexible docking, with the conformation with the lowest binding energy selected for plotting. Epimedin A1 binds an interdomain interface bridging the catalytic domain and membrane‐binding domain of the 5‐LOX protein, suggesting that it may induce conformational changes in 5‐LOX, thereby regulating its catalytic activity (Figure 7F). Epimedin A1 interacts with the amino acid residues Asp166, Trp102, and Arg68 of 5‐LOX through hydrogen bonds, and with His130 through an ionic bond (Figure 7G). We conducted cellular thermal shift assays to investigate if epimedin A1 directly binds to 5‐LOX, and used co‐treatment with the 5‐LOX selective inhibitor Zileuton to assess its impact on RvD5 biosynthesis. Epimedin A1 significantly enhanced the thermal stability of 5‐LOX protein in M2 macrophages, with a statistical difference above 52°C, suggesting that epimedin A1 may directly bind to 5‐LOX (Figure 7H,I). Co‐treatment with Zileuton (an inhibitor of 5‐LOX, 1 µm) significantly attenuated the promoting effect of epimedin A1 (3 µm) on RvD5 biosynthesis in M2 macrophages (Figure 7J). These results suggest that epimedin A1 may allosterically inhibit 5‐LOX to alter its catalytic activity, and promote RvD5 biosynthesis in M2 macrophages.

### 3.8 Epimedin A1 Inhibits Neutrophil Infiltration in the Colonic Mucosal Epithelium and Ameliorates DSS‐Induced Colitis in Mice

**FIGURE 8:**
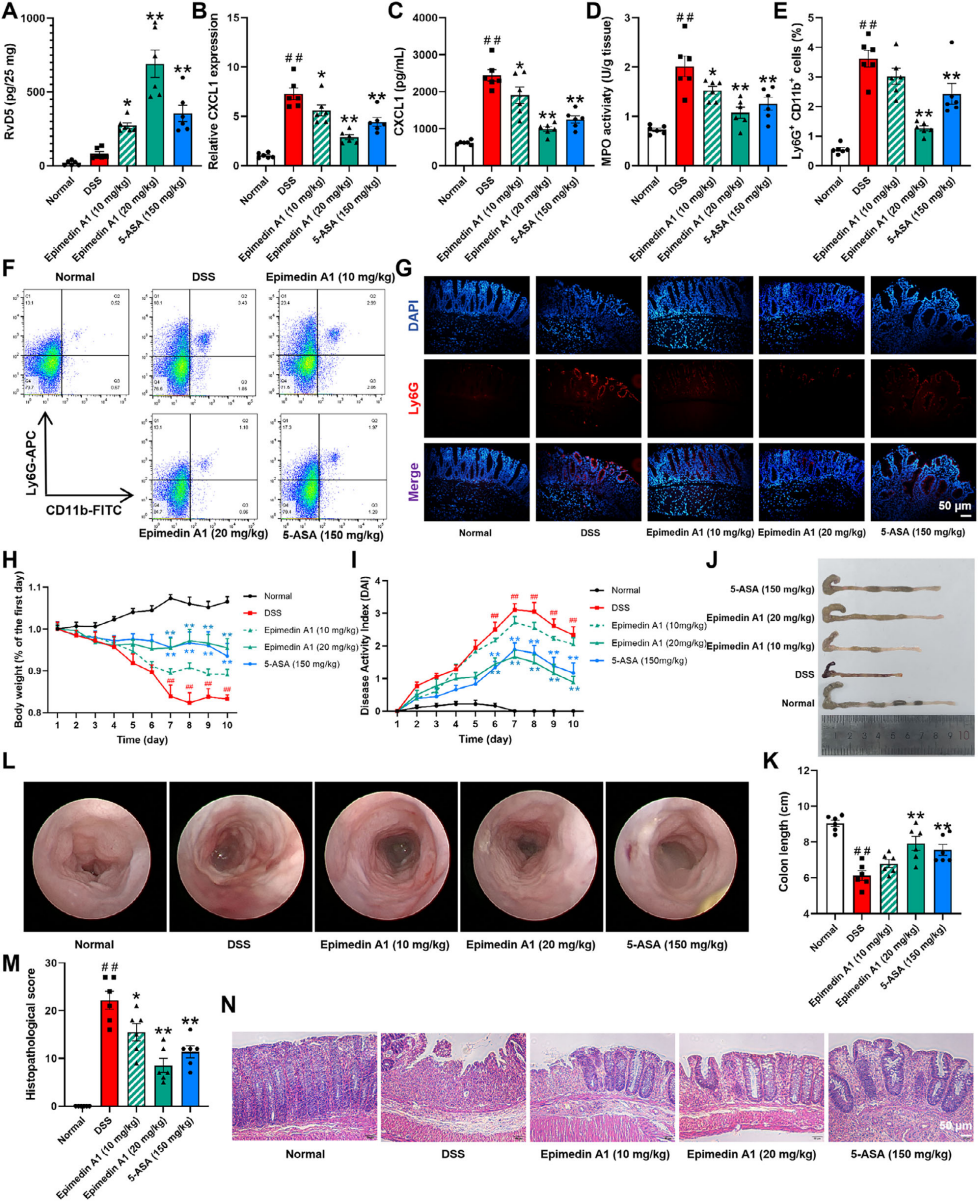
Attenuation of epimedin A1 on neutrophil infiltration in the colonic mucosal epithelium and colonic inflammation of mice with DSS‐induced colitis through the promotion of RvD5 biosynthesis. An experimental colitis model was induced in mice by administering 2.5% DSS in drinking water for 7 consecutive days, followed by oral administration of epimedin A1 (10, 20 mg/kg) and 5‐ASA (150 mg/kg) for 10 consecutive days. (A) The content of RvD5 in colon tissues was detected by UPLC‐MS/MS. (B, C) Primary colonic epithelial cells were isolated, and CXCL8 expression was detected by Q‐PCR and ELISA. (D) MPO activity in colons was detected by commercial kits. (E, F) Percentage of Ly6G^+^CD11b^+^ cells in the mononuclear cells of epithelium from mouse colon tissues was detected by flow cytometry. (G) Representative immunofluorescence images (scale bar, 50 µm). (H) Percentage change in body weight. (I) DAI score. (J, K) Colon length. (L) Representative images of endoscopy. (M, N) Pathological score was assessed by H&E‐stained (scale bar, 50 µm). Data are presented as the mean ± S.E.M. of six mice per group. ^##^ *P* < 0.01 vs the normal group; ^*^ *P* < 0.05, ^**^ *P* < 0.01 vs the DSS group.

To investigate the in vivo effects of epimedin A1 on RvD5 biosynthesis and neutrophil infiltration in the colonic mucosal epithelium of colitis mice, an experimental colitis model was established in mice with 2.5% DSS solution. Mice were orally administered with epimedin A1 (10, 20 mg/kg) and the positive control drug 5‐ASA (150 mg/kg) for 10 consecutive days. UPLC‐MS/MS technology was used to detect the RvD5 content in the colonic tissue of colitis mice. The results showed that epimedin A1 (10, 20 mg/kg) significantly increased the level of RvD5 in the colonic tissue (Figure 8A). Epimedin A1 (10, 20 mg/kg) and 5‐ASA (150 mg/kg) significantly downregulated the expression of CXCL1 in the primary colonic epithelial cells, with the effect of epimedin A1 (20 mg/kg) being superior to that of 5‐ASA (150 mg/kg) (Figure 8B,C). Epimedin A1 (10, 20 mg/kg) and 5‐ASA (150 mg/kg) significantly reduced the MPO activity in colonic tissue of colitis mice (Figure 8D). Epimedin A1 (20 mg/kg) and 5‐ASA (150 mg/kg) significantly decreased the proportion of neutrophils in the colonic mucosal epithelium of colitis mice, with the effect of epimedin A1 (20 mg/kg) being superior to that of 5‐ASA (150 mg/kg) (Figure 8E,F). Immunofluorescence results showed that epimedin A1 (10, 20 mg/kg) and 5‐ASA (150 mg/kg) significantly reduced the number of neutrophils in the colonic mucosal epithelium (Figure 8G). These results suggest that epimedin A1 effectively inhibits neutrophil infiltration in the colonic mucosal epithelium of DSS‐induced colitis mice.

Epimedin A1 (20 mg/kg) and 5‐ASA (150 mg/kg) significantly improved the weight loss of colitis mice (Figure 8H). Epimedin A1 (20 mg/kg) and 5‐ASA (150 mg/kg) significantly improved the symptoms of diarrhea, rectal bleeding, weight loss, and reduced the DAI scores of colitis mice, with the effect of epimedin A1 (20 mg/kg) being superior to that of 5‐ASA (150 mg/kg) (Figure 8I). Epimedin A1 (20 mg/kg) and 5‐ASA (150 mg/kg) significantly inhibited the colonic shortening induced by DSS in colitis mice (Figure 8J,K). Compared to the DSS group, epimedin A1 (10, 20 mg/kg) and 5‐ASA (150 mg/kg) significantly improved the colonic mucosal damage in colitis mice (Figure 8L). Epimedin A1 (10, 20 mg/kg) and 5‐ASA (150 mg/kg) significantly improved the colonic tissue pathological changes in colitis mice, with the effect of epimedin A1 (20 mg/kg) being superior to that of 5‐ASA (150 mg/kg) (Figure 8M,N). In conclusion, epimedin A1 can effectively improve colonic mucosal damage and inflammatory cell infiltration in DSS‐induced colitis mice.

## 4 Discussion

UC is a chronic inflammatory disease characterized by chronic intestinal inflammation and mucosal ulceration, clinically presenting symptoms such as diarrhea, abdominal pain, rectal bleeding, and weight loss. SPMs play a crucial role in promoting inflammation resolution and maintaining intestinal homeostasis. Clinical studies have found a significant decrease in RvD5 levels during active phase of UC, suggesting its key role in the resolution of intestinal inflammation. The present study demonstrated that RvD5, injected intraperitoneally, significantly alleviated colonic inflammation and mucosal tissue damage in mice with DSS‐induced colitis, suggesting that RvD5 has potentials to treat UC.

Neutrophils play a crucial role in the onset and progression of UC. When neutrophils infiltrate the mucosal epithelium, they exert potent pro‐inflammatory effects, disrupt the integrity of the epithelial barrier, and promote the recruitment of additional immune cells. Therefore, the effect of RvD5 on neutrophil infiltration in the colonic mucosal epithelium and lamina propria was investigated. The results showed that RvD5 specifically inhibited neutrophil infiltration in the mucosal epithelium. Given RvD5's properties as an immune modulator, it may effectively limit excessive neutrophil infiltration into the mucosal epithelium without compromising the normal antimicrobial functions of neutrophils, thereby reducing epithelial barrier damage. Clinical studies have shown that patients with persistent neutrophil infiltration in the colonic mucosal epithelium often exhibit resistance to anti‐TNF‐α therapy [11]. This discovery not only supports RvD5 as a supplementary therapeutic option for TNF‐α resistant patients but also provides new insights and directions for precision therapy in UC.

In the early stage of intestinal inflammation, neutrophils are quickly recruited from the microcirculation to the intestine, which is regulated primarily by chemokines [33]. Among them, CXCL8 (also known as IL‐8), released by intestinal epithelial cells, is widely recognized as a key factor in attracting neutrophils to the basolateral surface of the epithelium [34, 35]. In mice with colitis, CXCL1, a homolog of human CXCL8, is similarly considered a critical factor in early neutrophil chemotaxis [36]. Notably, mRNA and protein expression of CXCL8 in the intestinal mucosa of IBD patients are significantly increased [37]. Other epithelial‐derived chemokines like CXCL10, CXCL7, and CCL20 also significantly recruit and infiltrate neutrophils during UC and experimental colitis [38, 39, 40, 41]. To explore the mechanism of RvD5, we examined its effect on chemokine expression in colonic epithelial cells. The results showed that RvD5 selectively downregulated CXCL8 expression, and overexpression of CXCL8 counteracted inhibition of RvD5 against neutrophil chemotaxis, confirming the crucial role of CXCL8 in attenuation of RvD5 against neutrophil infiltration and colitis.

G‐protein‐coupled receptors (GPCRs) form a large family of membrane proteins, the most abundant cell surface receptors, transducing extracellular signals and playing critical roles in physiological and pathological processes [42]. SPMs exert pro‐resolution effect primarily through activating specific GPCRs [43]. Identified receptors for RvD5 include GPR32 [16] and GPR101, [44] yet the specific receptor responsible for mediating its inhibitory effect on neutrophil chemotaxis remains unclear. In vitro, selective knockdown of GPR32 and GPR101 in colonic epithelial cells showed that RvD5 downregulated CXCL8 expression via GPR101 but not GPR32. Furthermore, RvD5 significantly upregulated intracellular cAMP levels in colonic epithelial cells, promoting PKA phosphorylation, indicating that its inhibition of CXCL8 expression and neutrophil chemotaxis is likely mediated via the activation of GPR101.

GPR101, an X‐linked receptor located on Xq26.3, has been found to alter phenotype and function upon conditional macrophage knockout, compromising pro‐resolving function [45]. Additionally, levels of RvD5n‐3 DPA are significantly reduced in inflammatory arthritis mice, leading to intestinal inflammation and barrier disruption. Intraperitoneal injection of RvD5n‐3 DPA alleviates arthritis and intestinal inflammation in mice, restoring barrier function and reducing inflammation cell infiltration in paws [46]. Furthermore, GPR101 knockout significantly attenuates the inhibitory effects of RvD5n‐3 DPA on arthritis and intestinal inflammation in mice [40]. However, the role of GPR101 in the onset and progression of colitis remains unclear. Analysis of RNA‐seq data from the GSE107499 dataset revealed that the mucosal tissues of UC patients exhibited significantly downregulated expression of GPR101, and the expression levels of GPR101 were negatively correlated with neutrophil infiltration, suggesting that inadequate GPR101 activation in UC patients' mucosal tissues may be closely related to disease development.

Targeted regulation of CXCL8 expression can be achieved by inhibiting specific kinases or CXCL8 transcription factors [47]. In this study, we predicted 28 potential transcription factors for CXCL8 through GeneCards, humanTFDB, and PROMO databases, and analyzed their expression in mucosal tissues of UC patients and the correlation with GPR101. The results showed significantly reduced expression of STAT1 in high GPR101‐expressing colonic mucosal tissues of UC patients. Under TNF‐α stimulation, we validated this observation and found a significant upregulation of STAT1 expression in colonic epithelial cells, whereas RvD5 effectively suppressed STAT1 expression.

STAT1, a member of the signal transducers and activators of transcription (STAT) family, is activated by JAK kinases, which recruit and phosphorylate cytoplasmic STAT proteins, leading to nuclear translocation and transcription factor activity [48]. Abnormal JAK/STAT signaling upregulates pro‐inflammatory cytokine expression, disrupting immune balance and exacerbating the development of autoimmune diseases [49]. To verify if RvD5's inhibition of CXCL8 expression depends on downregulation of STAT1 expression, we constructed a STAT1 overexpression plasmid and transfected it into colonic epithelial cells, examining the effect on RvD5's inhibition of CXCL8 expression. Results showed that STAT1 overexpression significantly attenuated RvD5's inhibition of CXCL8 expression, indicating that STAT1 may be a key transcription factor for RvD5's inhibition of CXCL8 expression via GPR101 activation.

Our study showed that RvD5 significantly downregulates CXCL8 expression in colonic epithelial cells and effectively inhibits neutrophil infiltration into the colonic mucosal epithelium, suggesting it as a promising candidate for UC therapy. However, SPMs are rapidly metabolized and deactivated in vivo, [29] highlighting the potential of therapeutic strategies promoting biosynthesis over exogenous RvD5 supplementation.

Through molecular docking simulations of interactions between 2560 natural compounds and 5‐LOX allosteric inhibition sites, we identified 10 active ingredients capable of promoting RvD5 biosynthesis. Using UPLC‐MS/MS technology, we demonstrated that epimedin A1 significantly increased RvD5 levels in M2 macrophages, suggesting its role in promoting RvD5 biosynthesis. Epimedin A1, a major active ingredient in *Epimedium brevicomu* Maxim of the Berberidaceae family, is a flavonoid compound. Total flavonoids, the main active ingredients of *E. brevicomu*, have pharmacological effects such as anti‐aging, anti‐inflammatory, anti‐osteoporosis, antioxidant stress, and neuroprotection [50, 51]. We established a DSS‐induced mouse colitis model in mice, and epimedin A1 was orally administered to examine its effects on neutrophil infiltration in the colonic mucosal epithelium and its anti‐colitis effects. Results showed that epimedin A1 significantly inhibited neutrophil infiltration into the colonic mucosal epithelium of DSS‐induced colitis mice by upregulating the RvD5 level in colon tissue, effectively improving DSS‐induced colitis symptoms.

Although a proinflammatory microenvironment dominates during UC progression, previous studies have shown that the proportion of M2 macrophages does not necessarily decrease during colitis and can even increase during the remission or non‐active phase [52, 53, 54]. In our experimental design, Epimedin A1 was administered via gavage from the onset of the model, when GPR101 expression in epithelial cells had not yet been significantly reduced. Therefore, Epimedin A1 could promote RvD5 biosynthesis by M2 macrophages at an early stage, activate GPR101 signaling in epithelial cells, and subsequently downregulate CXCL8 expression, leading to reduced neutrophil recruitment and protection of the epithelial barrier. Since GPR101 is mainly expressed in colonic epithelial cells, its apparent downregulation during inflammation likely reflects epithelial barrier disruption. By protecting the epithelial barrier, Epimedin A1 may help preserve GPR101 expression, maintaining the receptor population necessary for the anti‐inflammatory action. While exogenous RvD5 might act more efficiently in directly promoting resolution, Epimedin A1, as a natural small‐molecule compound, could provide a more stable and practical therapeutic option, particularly for maintaining mucosal homeostasis and preventing relapse in individuals prone to UC.

## 5 Conclusion

RvD5, through the activation of GPR101, inhibits neutrophil infiltration in the mucosal epithelium, thereby ameliorating UC. The mechanism can be summarized as follows: RvD5 activates GPR101, leading to the downregulation of STAT1 and CXCL8 expression, ultimately resulting in reduced neutrophil infiltration in the mucosal epithelium and improvement in UC symptoms. By elucidating the mechanism of RvD5 in improving UC from the perspective of neutrophil infiltration in the mucosal epithelium, this study provides novel insights into UC treatment strategies. Additionally, the identification of epimedin A1 as a medicinal ingredient that promotes RvD5 biosynthesis offers valuable guidance for its development and utilization.

## Author Contributions

Y.D., Y.X., and Z.W. designed and supervised the study. P.G., Y.G., and Y.Z. collected and analyzed the data. P.G., Y.F., J.Z., and Y.H. conducted animal, cell cultivation and mass spectrometry experiments. Y.D., Y.X., and P.G. wrote the manuscript. Y.D., Y.X., K. Y. and Y.G. contributed to text revision and discussion. Y.D. was responsible for the overall content of this study. All authors discussed the results and approved the manuscript.

## Conflicts of Interest

The authors declare no conflicts of interest.

## Supporting information




**Supporting File**: advs74255‐sup‐0001‐SuppMat.docx.

## Data Availability

The data that support the findings of this study are available from the corresponding author upon reasonable request.
