# Supplementary material for: Resolvin D5 Inhibits CXCL8 Expression in Colonic Epithelial Cells Through Activating GPR101 to Impede Neutrophil Recruitment and Consequently Alleviate Ulcerative Colitis
Source: Adv Sci (Weinh). 2026 Feb 3;13(21):e15176. doi: 10.1002/advs.202515176 (PMC13073316; doi:10.1002/advs.202515176)
Supplement: Supplementary file 1 — Supporting File: advs74255‐sup‐0001‐SuppMat.docx. [file ADVS-13-e15176-s001.docx]

**Supplementary Information**

**Resolvin D5 inhibits CXCL8 expression in colonic epithelial cells through activating GPR101 to impede neutrophil recruitment and consequently alleviates ulcerative colitis**

The supporting information includes the following contents:

- Supplementary figures
- Supplementary table
- **Supplementary Figures**
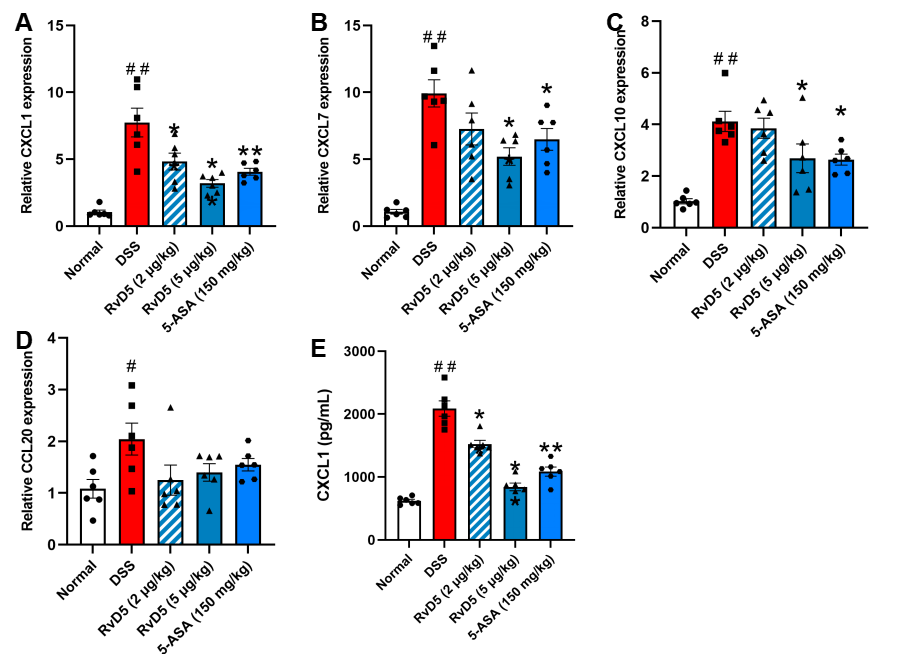


**Supplementary Fig. S1 Effect of RvD5 on the expression of neutrophil-associated chemokines in colonic epithelial cells of mice with DSS-induced colitis.** Colitis model was established in mice using DSS, and the mice were euthanized by cervical dislocation 1 h after the final dose. Colonic epithelial cells were isolated, and neutrophil-associated chemokine expression was assessed. (A-D) The mRNA expression of CXCL1, CXCL7, CXCL10, and CCL20 was detected by Q-PCR. (E) The protein expression of CXCL1 in colonic epithelial cells was detected by ELISA kit. Data are presented as the mean ± S.E.M. of six mice per group. ^#^*P* < 0.05, ^##^*P* < 0.01 *versus* the normal group; ^*^*P* < 0.05, ^**^*P* < 0.01 *versus* the DSS group.

**
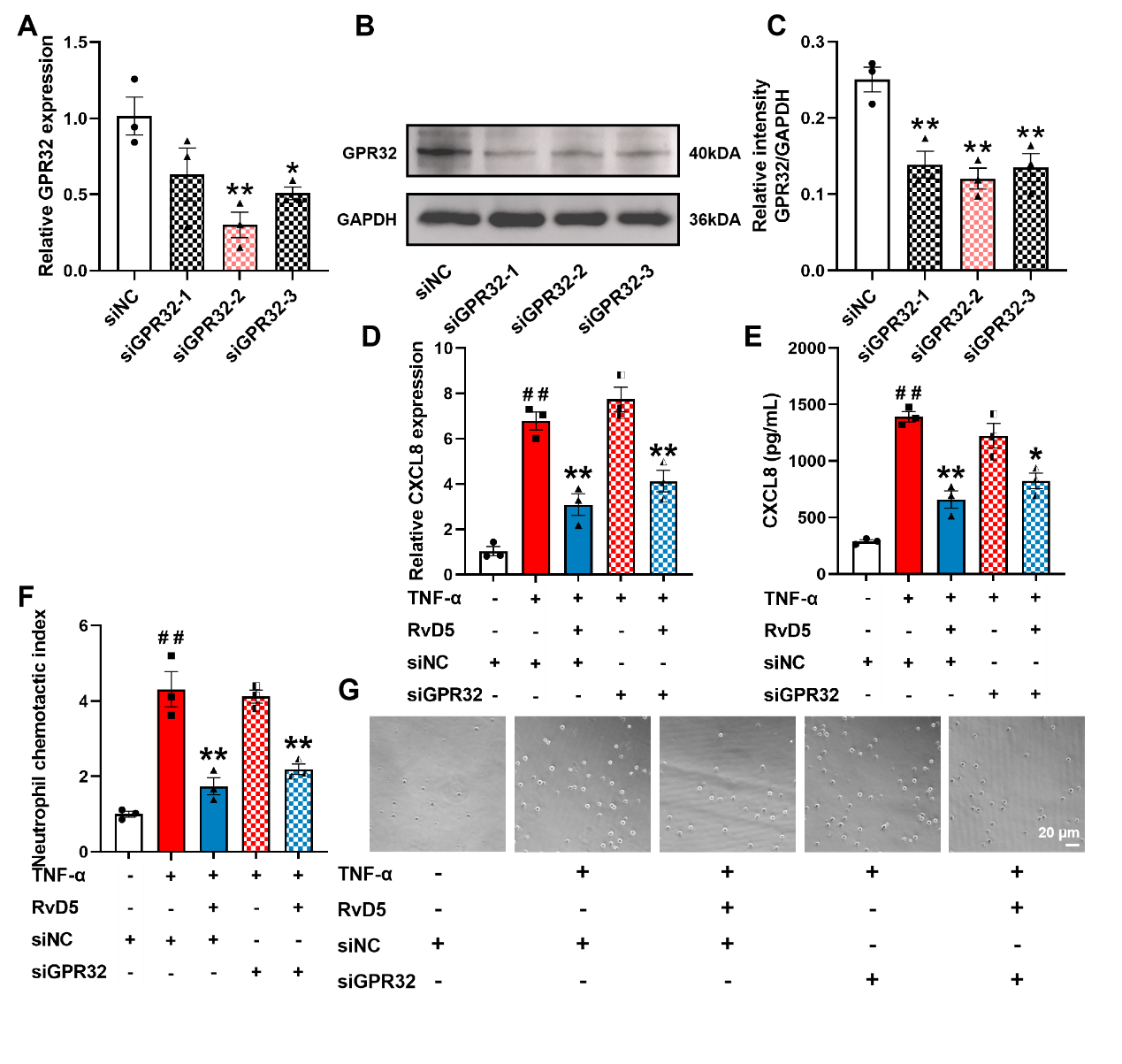
** **Supplementary Fig. S2 Insufficient GPR32 activation had no impact on the RvD5-mediated inhibition of CXCL8 expression and neutrophil chemotaxis towards colonic epithelial cells.** NCM460 cells were transfected with NC siRNA or GPR132 siRNA (siR-GPR32) for a duration of 72 h. (A) The knockdown efficiency was detected by Q-PCR detection. (B, C) The protein expression of GPR32 was detected by western blotting. (D) The mRNA expression of CXCL8 in NCM460 cells was detected by Q-PCR. (E) The protein expression of CXCL8 in NCM460 cells was detected by ELISA. (F, G) The chemotactic index of neutrophils towards NCM460 cells was detected by Transwell assay (scale bar, 20 μm). Data are presented as the mean ± S.E.M of three independent experiments. ^##^*P* < 0.01 *versus* the control group; ^*^*P* < 0.05, ^**^*P* < 0.01 *versus* the treatment group.


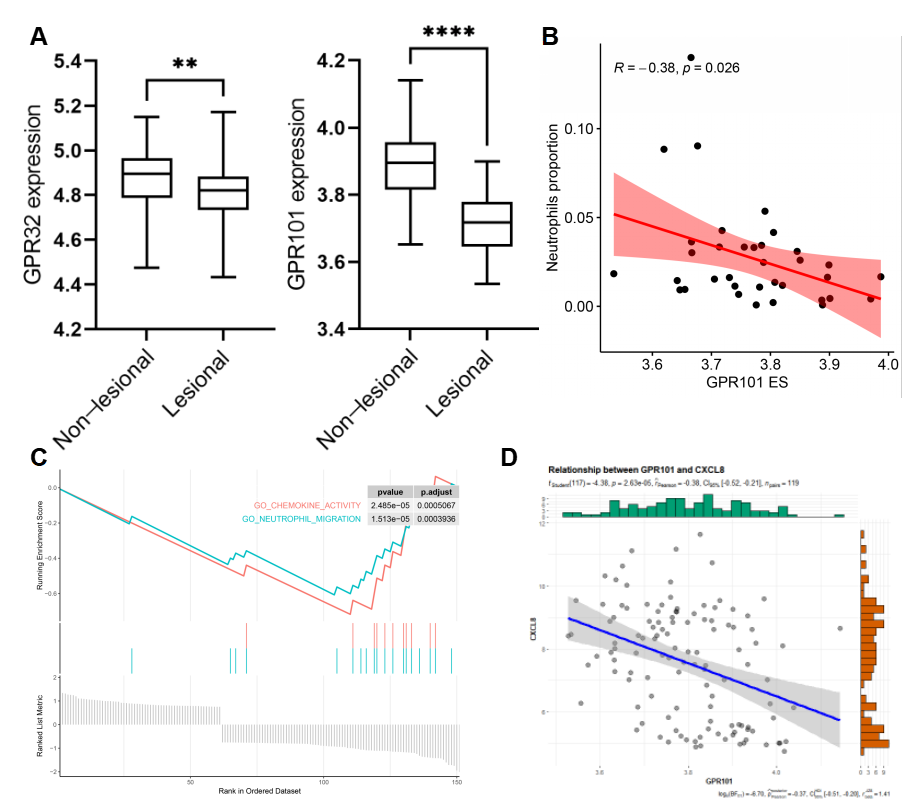
**Supplementary Fig. S3 The relationship between GPR101 expression in colonic mucosal tissue and neutrophil infiltration in UC patients.** RNA-seq data of mucosal tissues from UC patients were obtained from the GSE107499 dataset. The CIBERSORT algorithm was employed to quantify neutrophil proportions in mucosal tissue and establish their correlation with GPR101 expression. Enrichment analysis of GPR101-associated genes was performed using the GSEA package. (A)The mRNA expression levels of GPR101 in colonic mucosal tissues of UC patients. (B) Correlation analysis of neutrophil proportion and GPR101 mRNA expression. (C) GSEA analysis of different GPR101 expression groups in UC patients. (D) Correlation analysis of GPR101 and CXCL8 mRNA expression. The data are shown as mean ± S.E.M, and asterisks (^*^*P* <0.05, ^**^ *P* <0.01, ^***^ *P* <0.001, ^****^ *P* <0.0001) denote statistical significance.

**
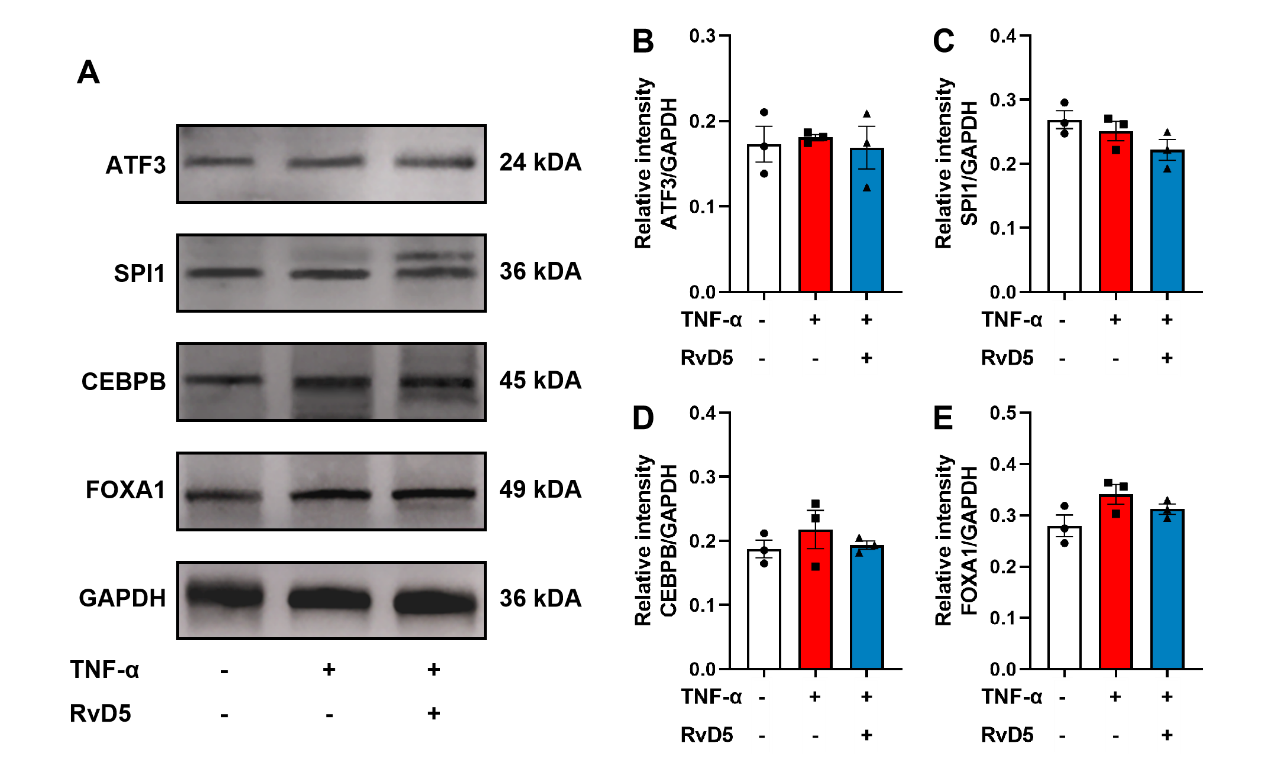
**

**Supplementary Fig. S4 Effect of RvD5 on ATF3, SPI1, CEBPB, and FOXA1 protein expression in NCM460 cells.** NCM460 cells were stimulated with TNF-α (50 ng/mL) and subsequently exposed to various concentrations of RvD5 (3 nM) for 24 h. (A-E) The protein expression of ATF3, SPI1, CEBPB, and FOXA1 was detected by western blotting. Data are presented as the mean ± S.E.M of three independent experiments. ^*^*P* < 0.05, ^**^*P* < 0.01 *versus* the control group.

**
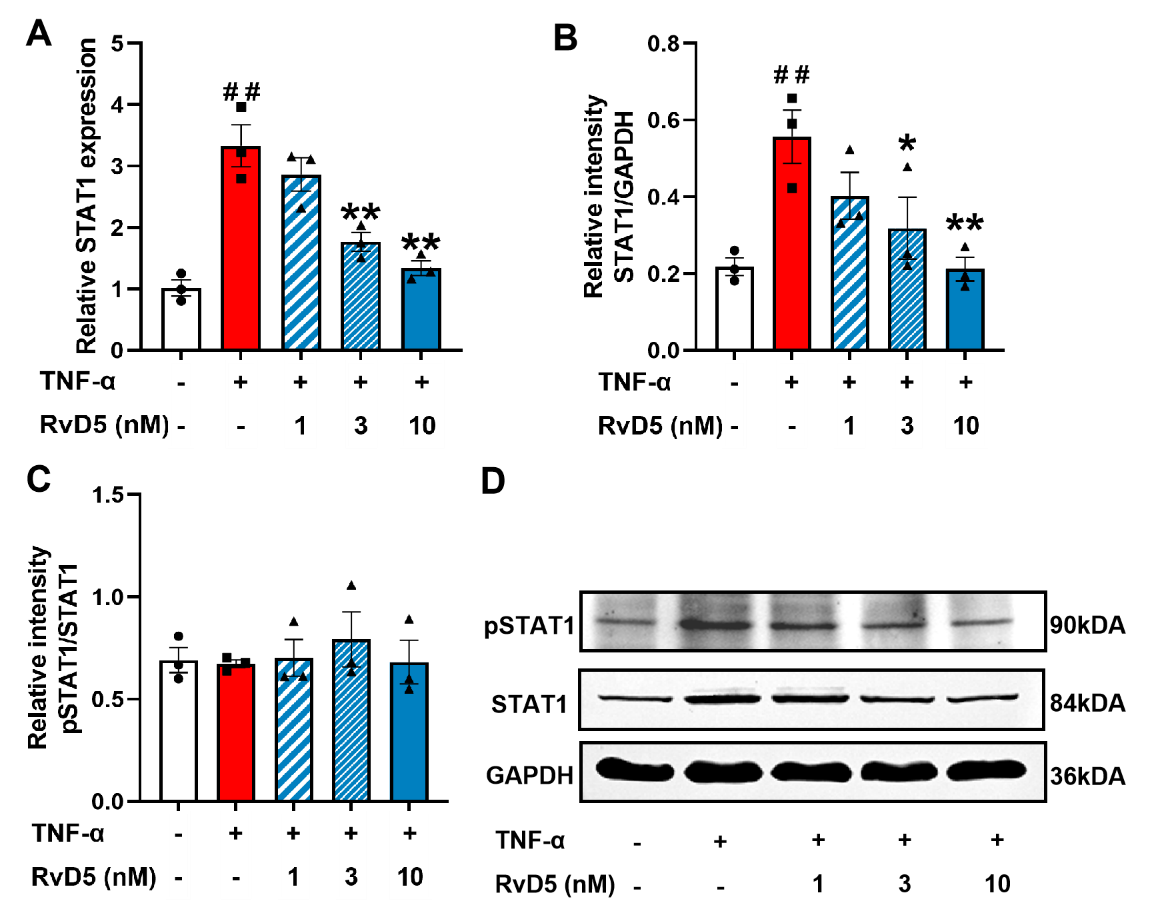
Supplementary Fig. S5 Effect of RvD5 on STAT1 expression in HT-29 cells.** HT-29 cells were stimulated with TNF-α (50 ng/mL) and subsequently exposed to various concentrations of RvD5 (1, 3, 10 nM) for 24 h. (A) The mRNA expression of STAT1 in HT-29 cells was detected by Q-PCR. (B-D) The protein expression of STAT1 and pSTAT1 was detected by western blotting. Data are presented as the mean ± S.E.M of three independent experiments. ^##^*P* < 0.01 *versus* the control group; ^*^*P* < 0.05, ^**^*P* < 0.01 *versus* the treatment group.


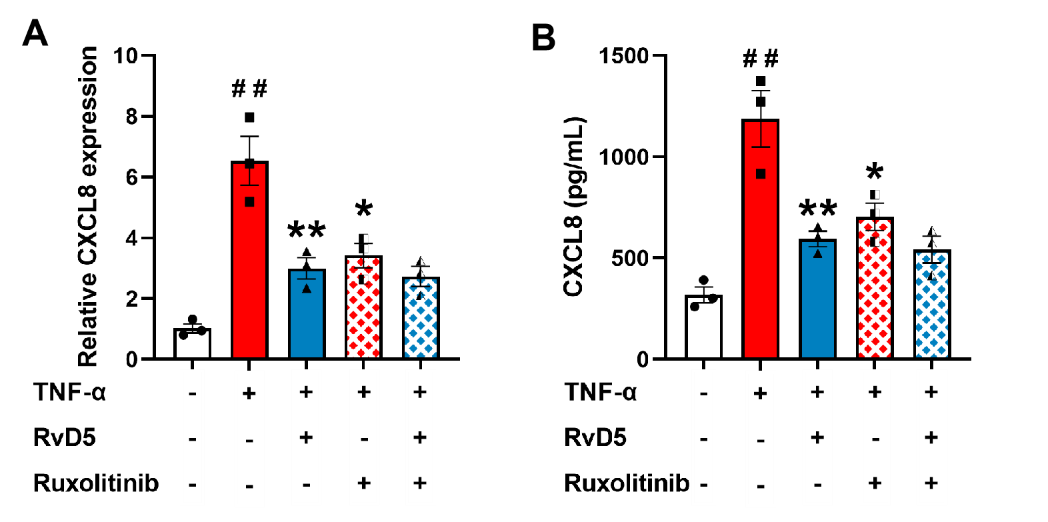


**Supplementary Fig. S6 Effect of Ruxolitinib on RvD5-mediated suppression of CXCL8 expression** **in NCM460 cells.** (A) The mRNA expression of GPR101 in colonic epithelial cells was detected by Q-PCR. (B) The protein expression of CXCL8 in colonic epithelial cells was detected by ELISA. Data are presented as the mean ± S.E.M of three independent experiments. ^##^*P* < 0.01 *versus* the control group; ^*^*P* < 0.05, ^**^*P* < 0.01 *versus* the treatment group.

**
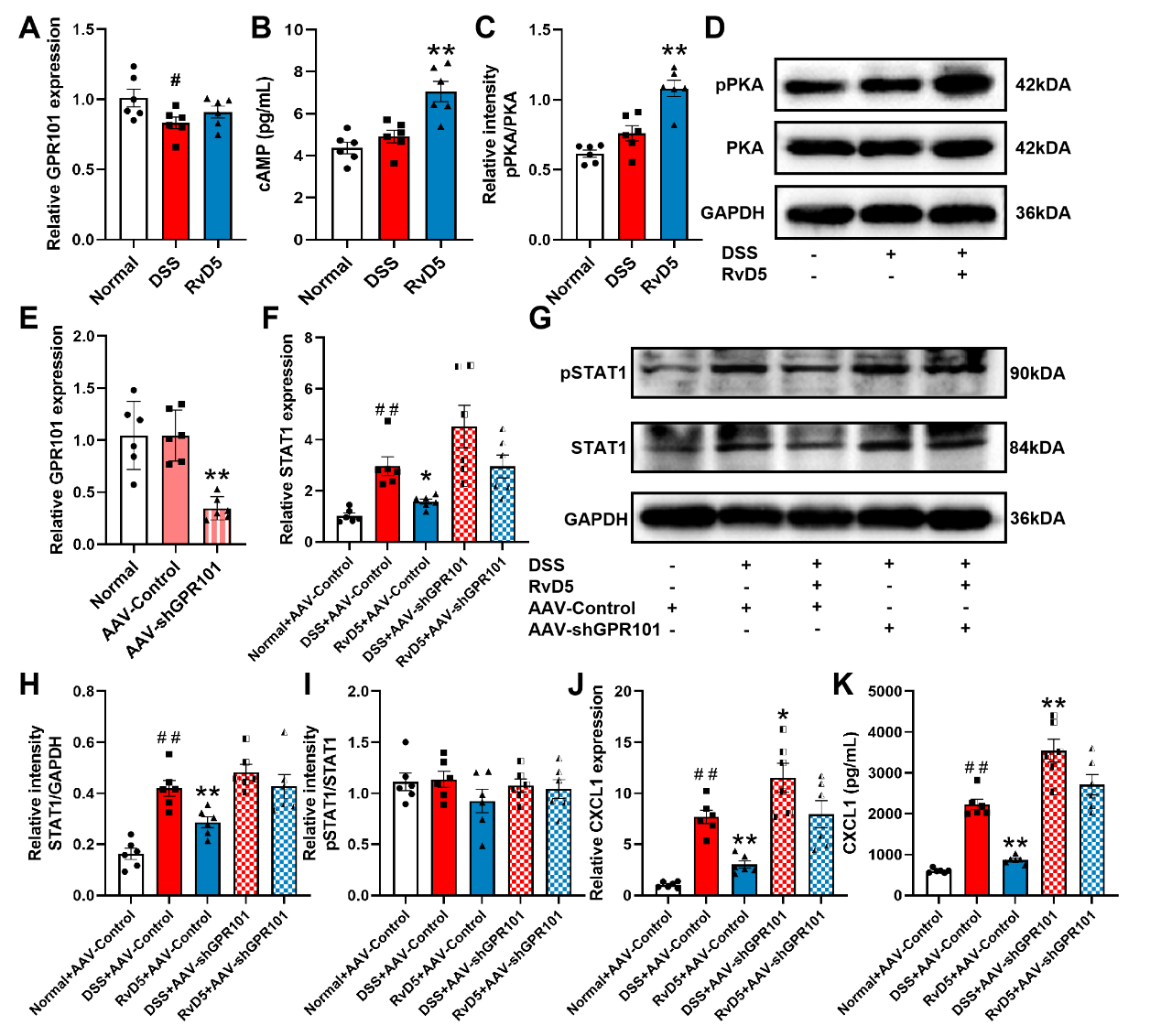
Supplementary Fig. S7 RvD5 activated GPR101 in colonic epithelial cells of DSS-induced colitis mice, leading to the downregulation of transcription factor STAT1 expression and CXCL8 expression.** (A) The mRNA expression of GPR101 in colonic epithelial cells was detected by Q-PCR. (B) Intracellular cAMP levels in colonic epithelial cells were detected by ELISA kit. (C, D) The protein expression of PKA and pPKA in colonic epithelial cells was detected by western blotting. (E) The knockdown efficiency was detected by Q-PCR detection. (F) The mRNA expression of STAT1 in colonic epithelial cells was detected by Q-PCR. (G-I) The protein expression of STAT1 and pSTAT1 in colonic epithelial cells was detected by western blotting. (J) The mRNA expression of CXCL8 in colonic epithelial cells was detected by Q-PCR. (K) The protein expression of CXCL8 in colonic epithelial cells was detected by ELISA. Data are presented as the mean ± S.E.M. of six mice per group. ^#^*P* < 0.05, ^##^*P* < 0.01 *versus* the normal group; ^*^*P* < 0.05, ^**^*P* < 0.01 *versus* the DSS group.

**
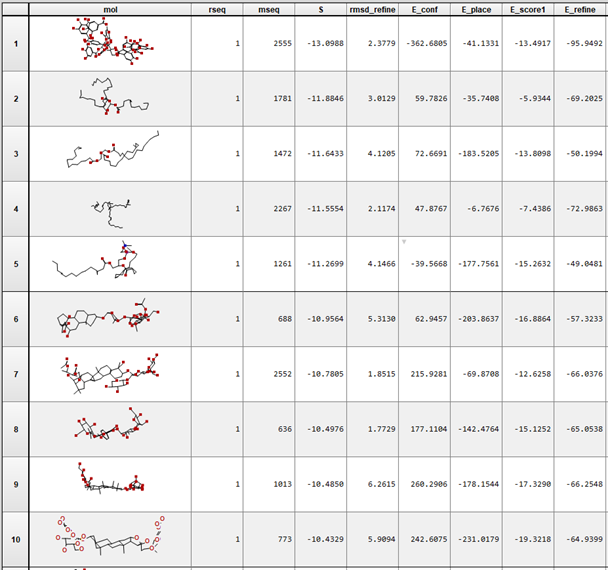
Supplementary Table S1** The top ten natural products identified through virtual screening.
